# Monooxygenase Activity of Indoleamine 2,3-Dioxygenase

**DOI:** 10.1021/jacs.5c17552

**Published:** 2026-02-05

**Authors:** Ali B. Lubis, Anna J. Bailey, Marko Hanževački, Christopher Williams, Mehul Jesani, Lola González-Sánchez, Christopher J. Arthur, Hannah C. Wilson, Andrea E. Gallio, Peter C. E. Moody, Matthew P. Crump, Adrian J. Mulholland, Allen M. Orville, Jonathan Clayden, Emma L. Raven

**Affiliations:** † School of Chemistry, 1980 University of Bristol Cantock’s Close, Bristol BS8 1TS, U.K.; ‡ Research Complex at Harwell, 120796 Harwell Science and Innovation Campus Didcot, Didcot, Oxfordshire OX11 ODE, U.K.; § Centre for Computational Chemistry, School of Chemistry, Cantock’s Close, University of Bristol, Bristol BS8 1TS, U.K.; ∥ Institute for Structural and Chemical Biology, Department Molecular and Cell Biology, 4488 University of Leicester, Leicester LE1 7RH, U.K.; ⊥ Department of Physical Chemistry, University of Salamanca, Salamanca 37008, Spain

## Abstract

Indoleamine 2,3-dioxygenase (IDO) is a heme-dependent enzyme that catalyzes the first, rate-limiting step of the kynurenine pathway—the oxidation of l-tryptophan to *N*-formylkynurenine (NFK). IDO-catalyzed depletion of tryptophan levels and accumulation of kynurenine pathway metabolites is an important control mechanism of the immune responses in cells. IDO has been considered as a dioxygenase because two atoms of oxygen are inserted into the substrate. Here, we use LC-MS and NMR to examine the reactivity of human IDO (hIDO) with l-tryptophan (l-Trp) and several other tryptophan analogues. Alongside dioxygenase activity, we identify a concurrent pathway of heme-dependent monooxygenase activity in the reaction of hIDO with l-Trp, leading to the formation of a cyclic 3a-hydroxy-1,2,3,3a,8,8a-hexahydropyrrolo­[2,3-*b*]­indole-2-carboxylic acid (HPIC) species. Reaction profiles for the reaction of hIDO with other tryptophan analogues are likewise examined. Formation of HPIC from l-Trp is reproduced in HeLa cells induced to overexpress hIDO, indicating that this dual dioxygenase/monooxygenase reactivity also occurs biologically. Notably, the reaction of hIDO with β-[3-benzo­(b)­thienyl]-l-alanine (S-l-Trp)—a known inhibitor —yielded only the cyclic HPIC analogue, suggesting that IDO activity can be selectively directed toward the monooxygenase pathway. Molecular dynamics simulations underscore the critical role of substrate plasticity within the active site of hIDO, while DFT calculations provide a mechanistic rationalization for the observed product distributions. Together, the data demonstrate dual dioxygenase/monooxygenase functionality for human IDO. As the overall gatekeeper for control of tryptophan levels in cells, the findings provide mechanistic information on relevance to therapeutic strategies focused on IDO inhibition.

## Graphical abstract

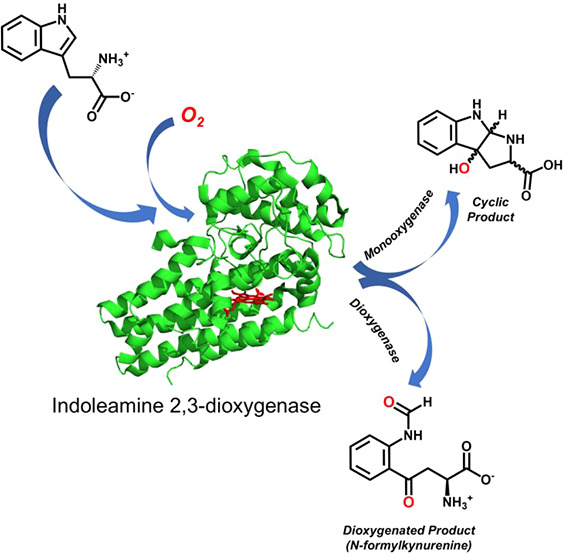

## Introduction

The kynurenine pathway—which leads to the formation of nicotinamide adenine dinucleotide NAD^+^—is the major mechanism for the metabolism of tryptophan in cells. Kynurenine pathway metabolites are important, as they are implicated in several diseases, such as Parkinson’s disease, Huntington’s disease, Alzheimer’s disease, and COVID-19.^1–8^ The first step of the kynurenine pathway is the oxidation of l-tryptophan and is catalyzed by two closely related O_2_-dependent heme-containing enzymes—indoleamine 2,3-dioxygenase (IDO) or tryptophan 2,3-dioxygenase (TDO). Once considered as a family of heme dioxygenase enzymes on its own, IDO and TDO are now classified as part of a larger superfamily of heme-dependent aromatic oxygenases.^9^ The product of the reaction, *N*-formylkynurenine (NFK, Scheme 1), is in turn metabolized to kynurenine and other downstream metabolites, leading ultimately to NAD^+^.^10,11^ Tryptophan dioxygenase activity was discovered in the 1930s,^12^ but it was not until the 1950s that the enzymes were purified^13–16^ and much later before the first structures appeared.^17,18^ Only quite recently did it become clear that control of tryptophan levels in cells is an important regulator of the immune system in both normal and disease biology. IDO in particular is known to be constitutively expressed in various tumors, and IDO-catalyzed tryptophan depletion is a mechanism by which tumor cells become invisible to the immune system and thus escape detection. As such, IDO has become a key focus for cancer immunotherapy,^19–24^ because controlling the oxidation of tryptophan and the formation of kynurenine pathway products would provide a mechanism for restoring normal immune function.

**Scheme 1:**
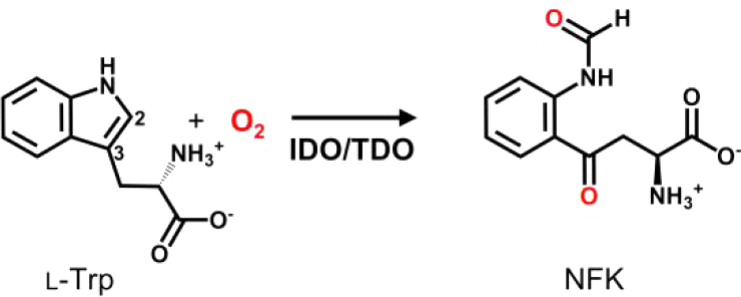
Reaction Catalyzed by IDO/TDO^a^

a Two oxygen atoms (red) are inserted into L-Trp to form NFK.

Formally, the process of tryptophan oxidation has been classified as heme-dependent aromatic dioxygenase activity because two atoms of oxygen, from O_2_, are inserted into the NFK product (Scheme 1). Early proposals for the mechanism of dioxygen atom insertion^25^ by IDO and TDO were later revised^26–36^ and a consensus emerged on the detailed steps involved.^29^ Like all heme enzymes that activate O_2_, the mechanism of tryptophan oxidation involves the formation of high oxidation states of iron. For most O_2_-activating heme enzymes, this occurs via the formation of a reactive Compound I intermediate which, in terms of electron count, is formally an Fe^V^=O species.^37^ The mechanism of tryptophan oxidation is different, however, in that a Compound II intermediate (formally an Fe^IV^=O species) is used instead of Compound I, so that dioxygen atom insertion is achieved directly from the ferrous-oxy state without the need for further reduction and leads to cleavage of the C^2^=C^3^ double bond of the substrate. The dioxygen atom insertion process is thus differentiated from the reactivity of the P450 (monooxygenase) enzymes—numerous P450s also react with indoles but typically carry out a hydroxylation reaction of a C–H bond by Compound I.^38^ The factors that differentiate these monooxygenase/dioxygenase reactivity differences are only partially understood.

Here, we demonstrate that IDO-catalyzed oxidation of l-Trp (or analogues of l-Trp) does not lead exclusively to the formation of NFK via a dioxygen atom insertion mechanism. Instead, we identify monooxygenase activity for IDO, which leads to the formation of cyclic species—3a-hydroxy-1,2,3,3a,8,8a-hexahydropyrrolo­[2,3-*b*]­indole-2-carboxylic acid (HPIC)—as a product, in some cases the exclusive product, of the reaction. We consider the implications of this dual reactivity profile in terms of heme-dependent IDO function and its biological role in cells.

## Results

### Enzyme-Catalyzed Oxidation of l-Trp and Substrate Analogues

Oxidation of a range of substrates (Scheme 2A) by ferrous human indoleamine 2,3-dioxygenase (hIDO) was examined (Figure 1).

**Scheme 2:**
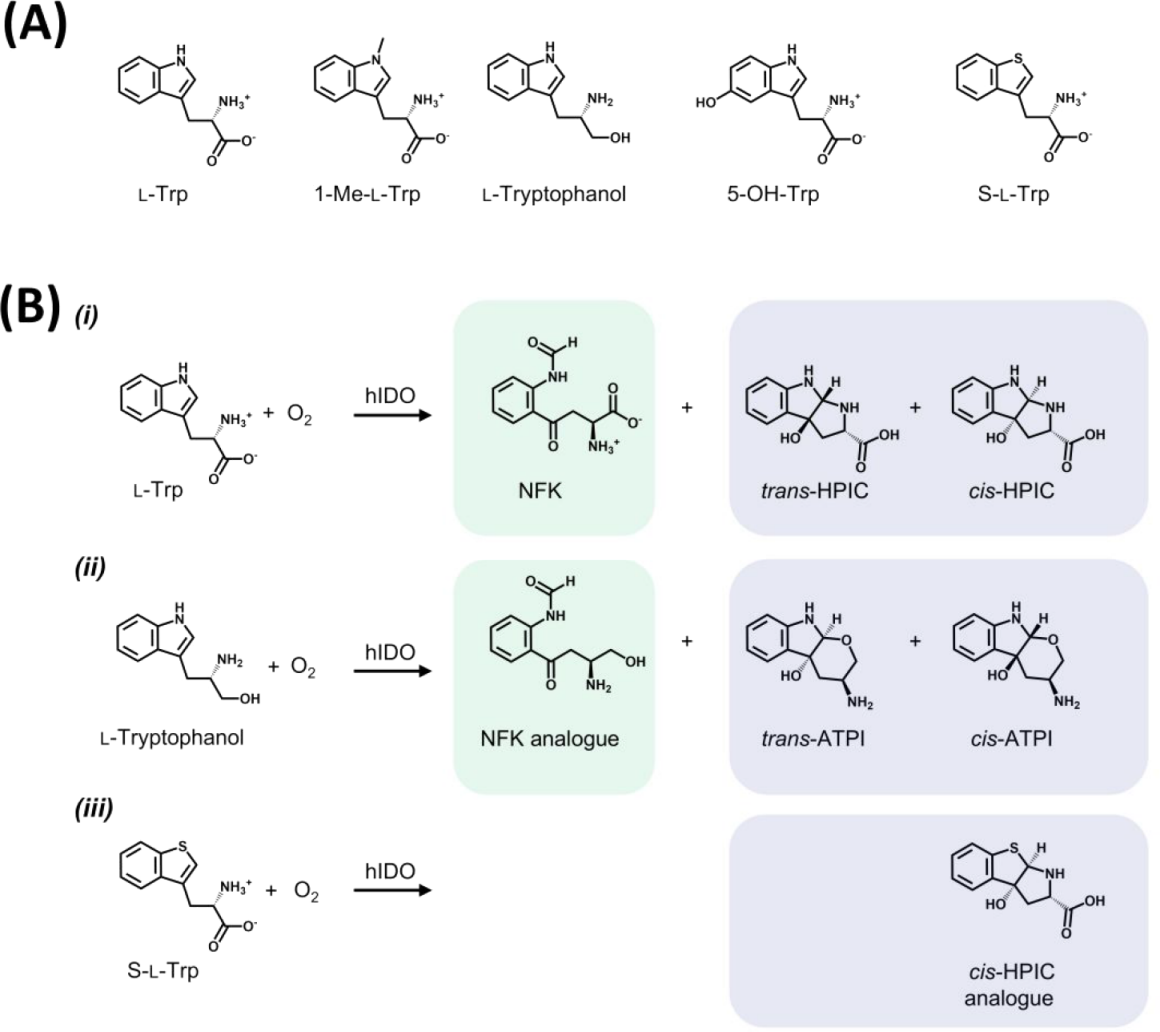
(A) Structures of Substrates Used in This Work. (B) Cyclic Products Observed on Reaction of hIDO with Various Substrates[Fn sch2-fn2]

a (i) l-Trp, leading to formation of NFK and *trans*- and *cis*-HPIC. (ii) l-Tryptophanol, leading to formation of the NFK analogue (*N*-(4-(2-aminophenyl)-1-hydroxy-4-oxobutan-2-yl)­formamide) and *trans*- and *cis*-ATPI (amino tetrahydropyranoindolol). (iii) S-l-Trp, leading to formation of the *cis*-HPIC analogue (hydroxy benzothieno pyrrole carboxylic acid)

**Figure 1:**
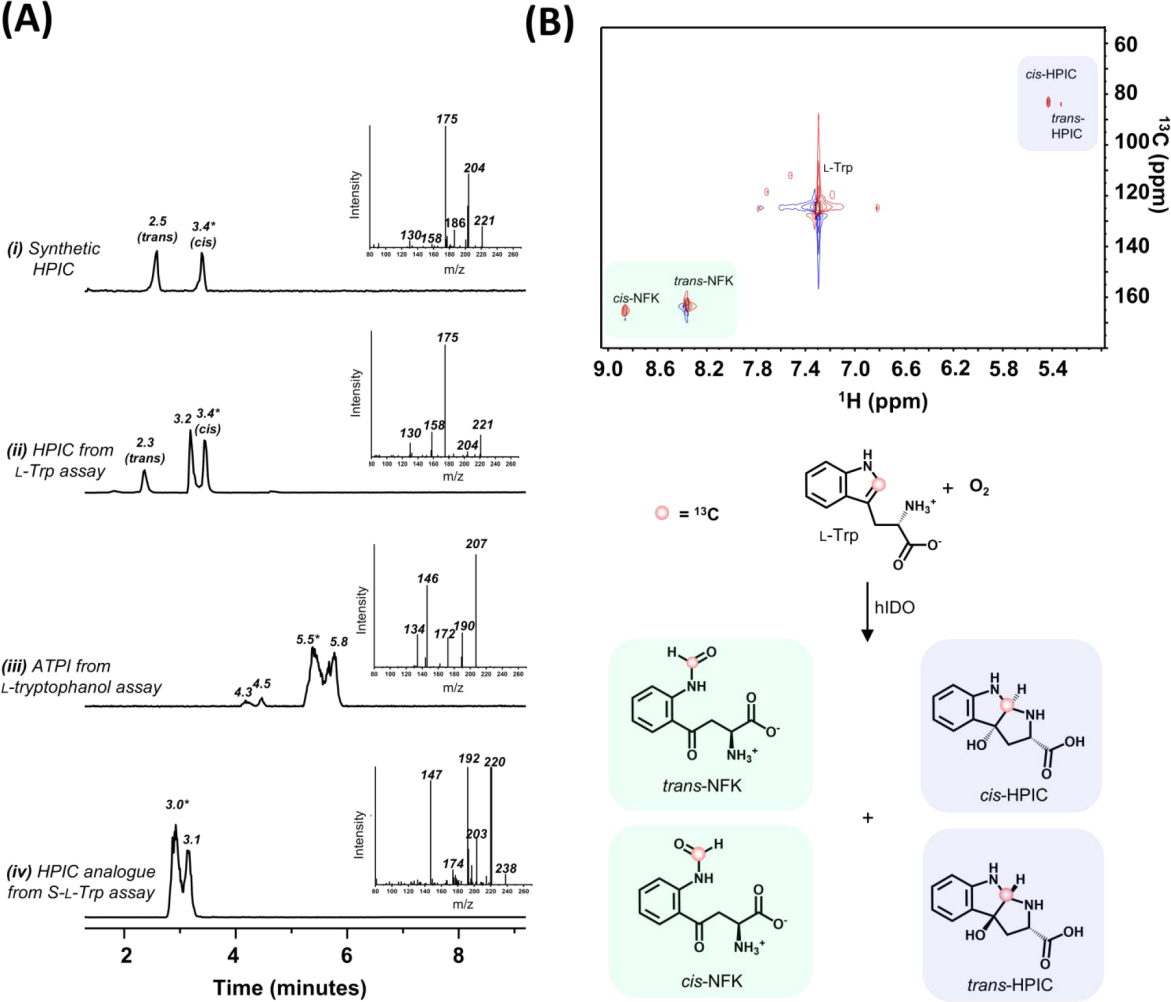
Enzymatic formation of cyclic products by hIDO. (A) LC-MS analyses in selected ion monitoring (SIM) mode of (i) synthetic HPIC and (ii)–(iv) products from reactions of hIDO with various substrates. (i) Elution profile for HPIC synthesized as described in Methods. Inset: mass spectrum of the *cis* isomer. (ii) Elution profile for HPIC generated from the reaction of hIDO with l-Trp. The additional peak at 3.2 min shows the same accurate mass and MS/MS fragmentation pattern as HPIC, but unequivocal assignment to a known HPIC stereoisomer was not possible. (iii) Elution profile for ATPI generated from the reaction of hIDO with l-tryptophanol. (iv) Elution profile for the cyclic HPIC analogue generated from the reaction of hIDO with S-l-Trp. Insets in (ii)–(iv) show the mass spectrum for the peak labeled * in each case. In (ii)–(iv), all the peaks in the LC analyses gave the same fragmentation pattern in the MS analyses as the peaks labeled *. (B) HSQC spectrum showing the ^13^C distribution observed on reaction of singly ^13^C-labeled l-Trp with hIDO; formation of *trans*- and *cis*-NFK, as well as *trans*- and *cis*-HPIC, is observed. Product profile from the reaction of singly ^13^C-labeled l-Trp with hIDO (the ^13^C atom is indicated with a red dot). Note that *trans*- and *cis*-NFK are rotamers, not geometric isomers, so they have different signals in ^1^H or ^13^C NMR but are not separable stereoisomers.

### l-Trp

Under enzymatic turnover conditions, human hIDO catalyzed the oxidative cleavage of l-Trp through the insertion of two atoms of oxygen derived from O_2_. Using LC-MS, two products were observed in these experiments. The first was NFK, which was confirmed by comparison with a sample of NFK that was synthesized chemically as described in the SI (Figure S1). Alongside the expected NFK product, a second product was identified, which was assigned as cyclic HPIC (Scheme 2B­(i)); the *trans* and *cis* isomers of HPIC elute separately at 2.3 and 3.2–3.4 min, respectively, with *m*/*z* = 221 (Figure 1A­(ii)). The product ratios are listed in Table 1. Analysis of the fragmentation pattern (Figures 1A­(ii) and S4) for the *cis*-HPIC peak, and comparison with the fragmentation pattern for a sample of HPIC that was synthesized chemically (Figures 1A­(i) and S3), confirmed that hIDO produces *cis-* and *trans-*HPIC during turnover. The mass fragmentation patterns of the two isomers are identical. Formation of *cis-* and *trans-*HPIC was also confirmed in the HSQC NMR spectrum of singly ^13^C-labeled l-Trp (Figure 1B). The ^13^C signal from l-Trp (at *ca* 120 ppm for ^13^C and 7.3 ppm for ^1^H) diverges to the cyclic product of *trans*-HPIC (80 and 5.3 ppm for ^13^C and ^1^H, respectively) and *cis*-HPIC (80 and 5.4 ppm, respectively), as well as *trans*-NFK (160 and 8.8 ppm, respectively) and *cis*-NFK (160 and 8.4 ppm, respectively). The signals for *trans*- and *cis*-NFK and *trans*- and *cis*-HPIC produced enzymatically are in agreement with those for products obtained synthetically (Figures S1D and S3E). Under the same turnover conditions, no reaction of hIDO with synthetic HPIC was observed, indicating that the cyclic product is a product of the enzymatic reaction (and not a substrate).

**Table 1:** Products and Product Ratios Observed during the Enzymatic Oxidation of l-Trp and Trp Analogues by hIDO

Substrate	Product ratio^a^
l-Trp^b^,^c^	84:4:12
1-Me-l-Trp	100:0:0
l-Tryptophanol	20:7:73
5-OH-Trp	100:0:0
S-l-Trp	0:0:100

a Product ratio (%) is expressed as (NFK: *trans*-HPIC/ATPI: *cis*-HPIC/ATPI).

b Under steady-state conditions, values of *k*_cat_ for the formation of NFK analogues for 1-Me-l-Trp and 5-OH-Trp are *ca* 2% of those for l-Trp (*k*_cat,Trp_ = 1.4 s^–1^).^39^ S-l-Trp^39^ and l-tryptophanol^40^ give no detectable change in absorbance at 321 nm in steady-state assays, so kinetic parameters cannot be obtained.

c Incorporation of ^18^O into HPIC has been previously confirmed^28^ in hIDO as well as in human TDO.

### 1-Me-l-Trp

To test whether the formation of HPIC, alongside NFK formation, is a more general feature of the hIDO mechanism, we examined the oxidation of several other substrates (Scheme 2). For 1-Me-l-Trp, further related LC-MS analyses confirmed the formation of *N*-formyl-methylkynurenine (Me-NFK, Figure S2 ii), and the corresponding fragmentation pattern in the mass spectrum confirmed the identity of the Me-NFK product (Figure S2 ii, inset). The corresponding HPIC product is not observed, however, in the reaction of hIDO with 1-Me-l-Trp (Table 1).

### l-Tryptophanol

hIDO-catalyzed oxidation of l-tryptophanol (Scheme 2) produces a different elution profile (Figure 1A­(iii)) from that of l-Trp, with a fragmentation pattern in the mass spectrum (Figures 1A­(iii) and S7) that is different from the HPIC product identified for l-Trp. The peak was identified as cyclic amino tetrahydropyranoindolol (ATPI) with *m*/*z* = 207, which is the major product (Table 1), with the NFK analogue (*N*-(4-(2-aminophenyl)-1-hydroxy-4-oxobutan-2-yl) formamide) as a minor product (Scheme 2B­(ii), Table 1 and Figure S2 iii). By analogy with the HPIC data in Figure 1A­(i), peaks at 4.3–4.5 min are assigned as the *trans* isomer, with the peaks at 5.5–5.8 min assigned as the *cis* isomer. ^1^H NMR spectra (Figure S11A) confirmed the emergence of new peaks consistent with the formation of the cyclic ATPI product.

### 5-OH-Trp

The pattern of product distribution for hIDO-catalyzed oxidation of 5-OH-Trp (Scheme 2A) is similar to that for 1-Me-l-Trp and gives the NFK analogue 5-OH-NFK (2-amino-4-(2-formamido-5-hydroxyphenyl)-4-oxobutanoic acid) as the only product (Table 1 and Figure S2iv); the corresponding fragmentation pattern in the mass spectrum confirmed the identity of 5-OH-NFK (Figure S2 iv, inset). No cyclic HPIC analogue is observed (Table 1) for hIDO-catalyzed oxidation of 5-OH-Trp.

### S-l-Trp

For hIDO-catalyzed oxidation of S-l-Trp, only the cyclic HPIC analogue with *m*/*z* = 238 is observed (Scheme 2B­(iii), inset and Figure 1A­(iv)) and was assigned as the *cis*-isomer (hydroxy-benzothieno-pyrrole carboxylic acid) by analogy to the data for synthetic HPIC (Figure 1A­(i)). The fragmentation pattern is consistent with that expected for the cyclic product (Figure S10). ^1^H NMR spectra (Figure S11B) confirmed the emergence of new peaks consistent with the formation of a cyclic product.

### Computational Analysis of Reaction Profiles

Molecular dynamics and DFT were used to rationalize the reactivities observed above.

#### S-l-Trp Reactivity

To gain a better understanding of the unique reactivity profile for S-l-Trp, extensive molecular dynamics (MD) simulations were performed (see SI for details). Analysis of the protein backbone root-mean-square deviation (RMSD) indicated that the protein maintained relative structural stability throughout the simulations, with maximum RMSD values remaining below 4 Å when compared with the reference crystal structure (Figure S12A). Notably, this stability was particularly evident upon the exclusion of residues 363–373, corresponding to the JK-loop, which is unresolved in the crystal structure due to its high flexibility,^41,42^ as supported by B-factor analysis (Figure S12B). Examination of the substrate positioning relative to the heme group and the bound O_2_ revealed distinct binding conformations for l-Trp and S-l-Trp. The C^2^ atom of the substrate was found to be closer to the heme-bound O_2_ in the l-Trp complex (3.2 Å) compared to the S-l-Trp complex (3.6 Å, Figure 2A). Both complexes exhibited similar interactions with the protein, as evidenced by the distances between the amine group of the substrate and the heme 6-propionate, as well as between the amine group of the substrate and the distal oxygen of the bound O_2_ (Figure 2A).

**Figure 2:**
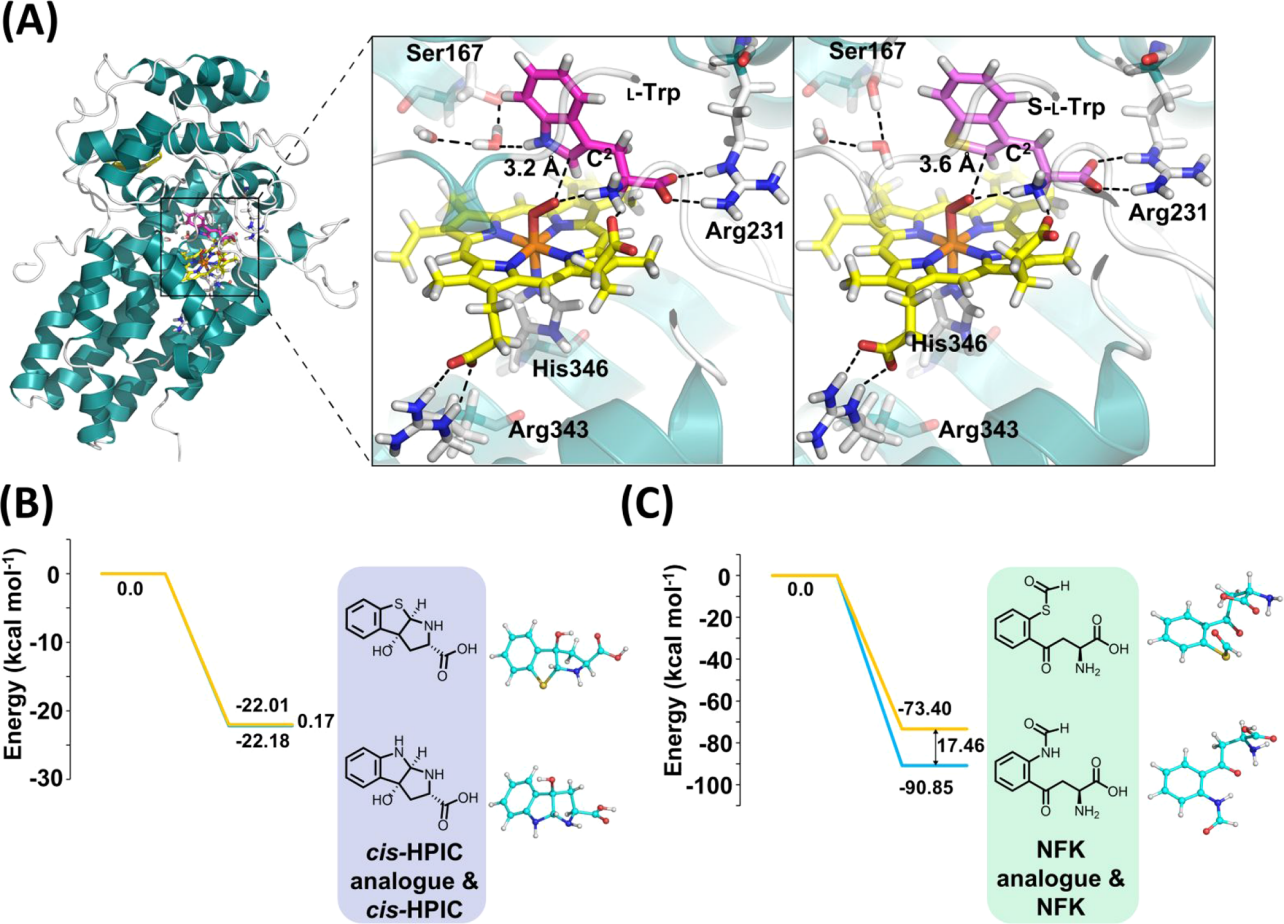
Computational analyses. (A) Representative MD snapshots of hIDO in complex with O_2_, l-Trp (left), and S-l-Trp (right). A key similarity in both structures is the position of oxygen bound to the heme, where the oxygen atom lies close to the amine group of both substrates and is oriented toward the C^2^ position of the substrate (see Figure S12C). The carboxylate group of each substrate also interacts with Arg231 in a similar manner. The amine group of both substrates forms similar hydrogen bonding interactions with the carboxylate of the 6-propionate (measured as the distance between the amine nitrogen atom and the carbonyl carbon atom of the propionate) and distal oxygen bound to the heme cofactor. The most notable difference lies in water molecule interactions: in the l-Trp complex, a water molecule forms a hydrogen bond with the NH group of the indole ring, whereas no such hydrogen bonding is observed with S-l-Trp. For atom labeling and the distribution of distances, see Figure S12C,D. Further differences are evident in the positions of specific atoms, as detailed in Figure S12D. (B) Relative energy diagram of *cis*-HPIC formation from the *cis*-epoxide intermediate in l-Trp and S-l-Trp. (C) Relative energy diagram of NFK formation from the *cis*-1,2-dioxetane intermediate during dioxygen atom insertion of l-Trp and S-l-Trp. The dioxetane intermediate (see Figure S12E for DFT structures) was used here to enable direct comparison of the relative energies without including the heme cofactor (see step 4b in Scheme 3C for the corresponding mechanistic rationale).

#### Product Distributions

To rationalize the observed product distributions, density functional theory (DFT) calculations were employed to investigate the energetics associated with the formation of the monooxygenated cyclic intermediate derived from the reactions of l-Trp, S-l-Trp, and l-tryptophanol. This included the experimentally observed cyclic *cis*-HPIC products from l-Trp and S-l-Trp, the putative but undetected NFK analogue from S-l-Trp, and the cyclic ATPI product formed from l-tryptophanol. The computed product energies for *cis-*HPIC derived from l-Trp and S-l-Trp were nearly identical, differing by only 0.17 kcal mol^–1^ (Figure 2B), indicating a similar thermodynamic feasibility for their formation. Binding interactions of *cis*- and *trans*-HPIC with IDO were also investigated by using protein–ligand docking. For l-Trp, *cis*-HPIC exhibited stronger binding compared to that of *trans*-HPIC (Table S1), with a more pronounced difference observed for S-l-Trp. These data suggest an inherent binding preference for *cis*-HPIC, which may explain the lack of *trans*-HPIC formation from S-l-Trp (Table S1, Figure S14). In contrast, NFK formation was significantly more favorable from l-Trp compared to S-l-Trp, with an energy difference of 17.46 kcal mol^–1^ (Figure 2C), consistent with the lack of NFK observed experimentally in the reaction with S-l-Trp (Table 1). This selectivity is attributed to differences in the electronic structure: while l-Trp enables resonance stabilization via a planar amide in NFK, the thioester moiety in S-l-Trp disrupts conjugation with the aromatic ring, rendering the analogous product thermodynamically less stable. Noncovalent interaction analysis revealed that the *trans*-HPIC isomer exhibits additional stabilization through favorable intramolecular van der Waals interactions between the aryl ring and carboxylic acid group, resulting in a slightly lower energy compared to that of *cis*-HPIC (Figure S13A). The DFT energy difference was below 1 kcal mol^–1^, which was generally considered to be quantitatively insignificant. These calculations were for isolated molecules and so do not include the effects of the environment. Notably, in all MD simulations of all substrates, the spatial positioning of the substrate C^2^=C^3^ double bond relative to the heme-bound O_2_ consistently favored *cis*-HPIC formation.

For the hIDO-catalyzed oxidation of l-tryptophanol, both the *cis-* and *trans-*isomers of the cyclic ATPI product (featuring two six-membered and one five-membered ring, Scheme 2B­(ii)) were calculated to be energetically more favorable than the alternative isomer with one six-membered and two five-membered rings (i.e., analogous to the HPIC scaffold observed from l-Trp oxidation, Figure S13B), further supporting the experimentally observed product selectivity.

The formation of *cis*-HPIC from 1-Me-l-Trp and 5-OH-Trp was calculated to be energetically similar to that observed for both l-Trp and S-l-Trp, suggesting that the products could, in principle, be formed (Figure S13C). The absence of HPIC-like products in experiments with these two substrates implies that factors other than thermodynamics control the reactivity. Specifically, steric hindrance between the methyl substituent in 1-Me-l-Trp (Scheme 2A) and the lone pair of the nitrogen group of the amine side chain may increase the activation barrier during ring cyclization, effectively preventing product formation. MD simulations revealed that the methyl group of 1-Me-l-Trp displaces water molecules from the active site, thereby enhancing hydrophobic interactions with the side chains of Ser167 and Ala264. Further DFT calculations comparing l-Trp and 1-Me-l-Trp (see SI and Figure S15) indicate that the indole nitrogen is less electron-rich in 1-Me-l-Trp and support a higher electron-donating capacity of N1 in l-Trp (compared to that in 1-Me-l-Trp). This diminishes electron donation to the C^2^ position of the substrate in 1-Me-l-Trp (step 3a, Scheme 3C), which is important for HPIC formation.

**Scheme 3:**
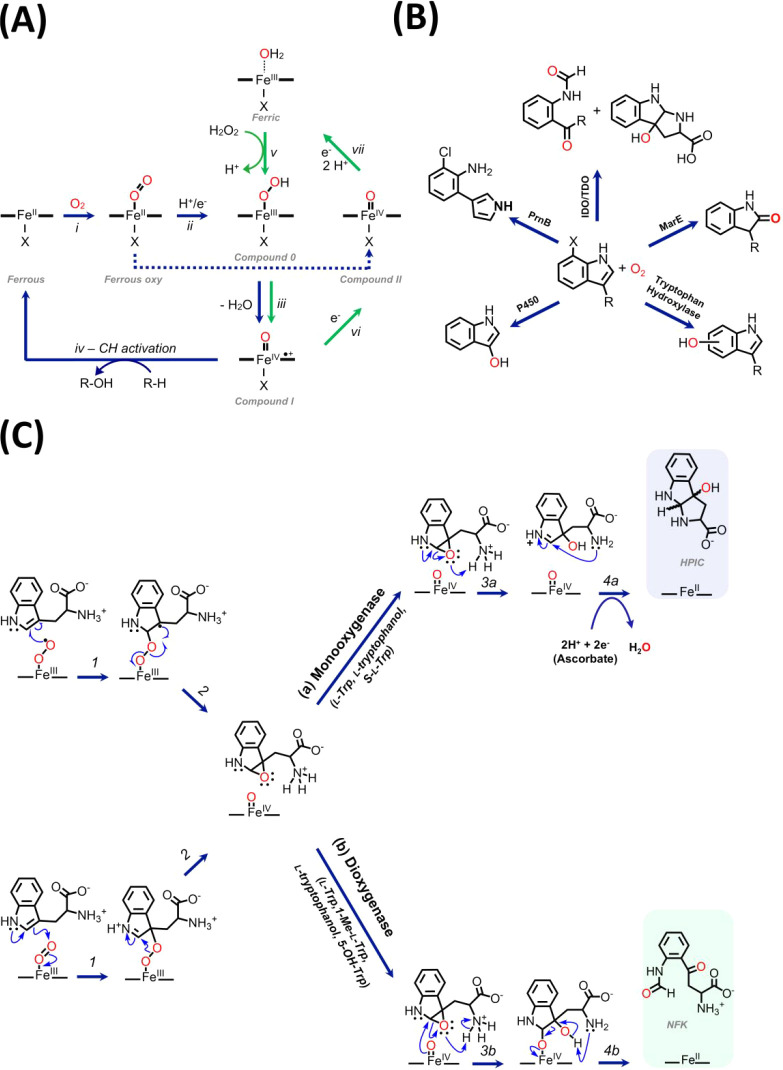
Mechanism of O_2_ Activation in Heme Enzymes[Fn sch3-fn3]

a (A) For some heme enzymes, O_2_ reacts with reduced heme (Fe^II^, step i, blue arrows), and further reduction (step ii) gives an unstable Fe^III^–OOH species, which decays rapidly (step iii) to a 2-electron oxidized intermediate (Compound I). In P450s, the cycle completes via C–H bond activation (step iv). In other heme enzymes, oxygen activation occurs by reaction with H_2_O_2_ (green arrows, step v) through the same Compound I species (step iii) and one-electron reduction by substrate (steps vi and vii, through Compound II). In IDO, oxygen activation occurs directly via Compound II (blue dotted arrow), bypassing Compound I. The proximal ligand (X) to the heme (black line) is usually a His (in IDO and tryptophan hydroxylase) or Cys (in P450s). (B) Comparison of reactions catalyzed by IDO/TDO, P450, tryptophan hydroxylase (TPH), MarE, and PrnB (R = −CH_2_CHNH_3_^+^COO^–^ for IDO and TPH, R = CH­(CH_3_)­CHNH_3_^+^COO^–^, R = H for P450; X = H for all substrates except for PrnB, where X = Cl). No hydroxylated products, analogous to those formed by TPH, were observed for hIDO. (C) Proposed mechanism for oxidation of l-Trp by hIDO, showing concurrent (a) monooxygenase and (b) dioxygenase pathways for formation of (a) the cyclic HPIC/ATPI products and (b) NFK (or NFK analogues). Efficient formation of HPIC in the monooxygenase pathway is dependent on the presence of a reductant (ascorbate in our experiments), although trace amounts of HPIC are observed in the absence of ascorbate. NADH as a reductant also supports HPIC formation (see SI)

Similarly, the 5-hydroxy group of 5-OH-Trp was frequently observed to form a hydrogen bond with the side chain of Cys129, which likely contributes to a more rigid substrate conformation, thereby limiting the conformational flexibility essential for efficient formation of the cyclic product (Figure S13D).

### Oxidation of l-Trp in HeLa Cells

HeLa cells express IDO inducibly, and IDO expression can be upregulated with interferon-γ (IFN-γ), forming the basis of the IDO-induced immune response in cancer cells. We therefore assessed HeLa cell cultures for formation of IDO-catalyzed oxidation. In HeLa cells induced with IFN-γ, induction of IDO-1 was confirmed (Figure S16). In these cells, we identified the formation of both NFK (Figure 3A) and *trans*- and *cis*-HPIC (Figure 3B). In samples collected from the growth medium of IFN-γ-induced HeLa cells supplemented with l-Trp, increased formation of *trans*- and *cis*-HPIC is observed (Figure 3B­(iii)), although the ratios of *trans*- and *cis*-HPIC differ slightly from those observed *in vitro* (Table 1).

**Figure 3:**
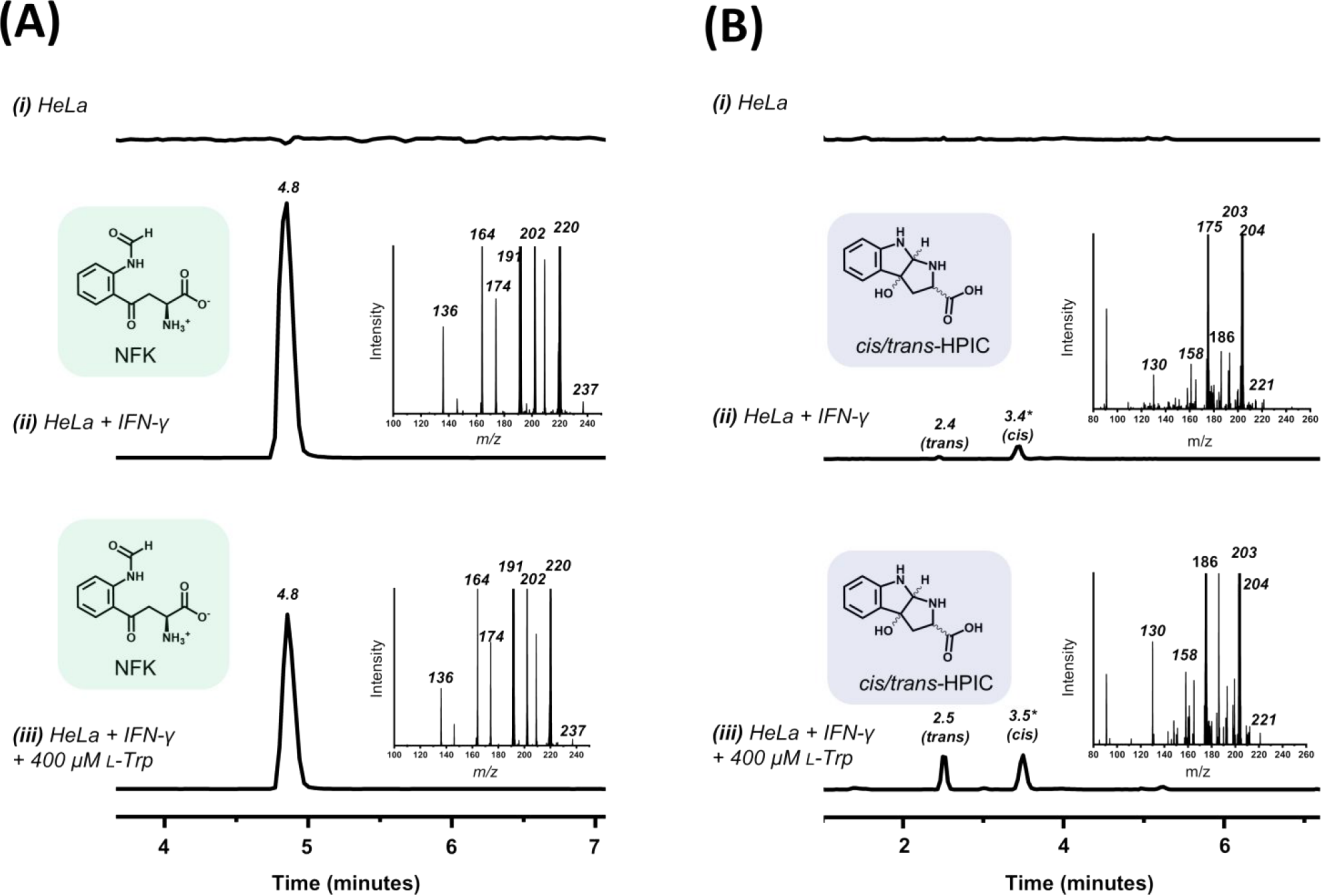
Oxidation of l-Trp in HeLa cells. Elution profiles in selected ion monitoring mode showing formation of (A) NFK and (B) *trans*- and *cis*-HPIC in (i) uninduced HeLa cells, (ii) HeLa cells induced with IFN-γ (5 ng/mL), and (iii) HeLa cells induced with IFN-γ and incubated with added l-Trp for 24 h prior to analysis. The corresponding mass spectra of (A) NFK (*m*/*z* = 237) and (B) HPIC (*m*/*z* = 221) are shown in the insets. Mass spectra for *trans*-HPIC, *cis*-HPIC, and NFK are all consistent with the data in Figures 1A­(i) and S2i.

## Discussion

All heme enzymes that activate dioxygen make use of the same basic mechanism and the same reaction intermediates to perform biological oxidations (Scheme 3A). They all react with either dioxygen (O_2_) or hydrogen peroxide (H_2_O_2_) to generate high-oxidation-state ferryl intermediates. These reactive ferryl species exist as either Compound I (formally an Fe^V^=O species) or Compound II (Fe^IV^=O) intermediates, and they provide the driving force for oxidative catalysis. Cytochrome P450 monooxygenase enzymes use a Compound I intermediate and use it to carry out oxygen atom insertion into C–H bonds. Hydroxylation of indoles is an example of such P450 reactivity (Scheme 3B). Heme-containing tryptophan dioxygenase enzymes, on the other hand, do not use Compound I as part of their mechanism (Scheme 3C)^36^ and yet are able to catalyze dioxygen atom insertion into an indoleamine-containing substrate (Scheme 1). The advantage of this mechanism is that, unlike the P450s, which need continuous rereduction for each enzymatic turnover, a single reducing equivalent is sufficient to initiate continuous turnover.

In this work, we identify a pathway for monooxygenase activity (single oxygen atom insertion) in an IDO that operates concurrently with the expected (NFK-forming) dioxygen atom insertion activity. Scheme 3C illustrates a mechanism to account for the bifunctional monooxygenase/dioxygenase reactivity, with both pathways operating through an initial ferrous-oxy species and Compound II. Support for this mechanism comes from the fact that Compound II is an observed intermediate in the reactions of IDO with l-Trp, 1-Me-l-Trp, S-l-Trp, and 5-OH-Trp.^39^ For l-Trp, l-tryptophanol and S-l-Trp, both the (a) and (b) pathways are used. For S-l-Trp, only the cyclic product (pathway a) is observed, which is consistent with kinetic data that identify S-l-Trp as an inhibitor;^40^ for 1-Me-l-Trp and 5-OH-Trp, only NFK (pathway b) is observed. Formation of cyclic products for l-tryptophanol and S-l-Trp is proposed as occurring through a mechanism similar to that shown in Scheme 3C, Figure S17. The presence of a reducing agent is required for the monooxygenation process (Scheme 3C, step 4a), which depends on rereduction of the iron for turnover and release of the ferryl oxygen as water. In this *in vitro* experiment, the reducing agent comes from ascorbate, but NADH was also found to support HPIC formation and may replicate the overall reducing environment present in cells. This is in contrast to the dioxygen atom insertion steps leading to the formation of NFK, which do not require electrons for turnover after the initial reduction of the heme.

That IDO uses Compound II, and not Compound I as in the P450s, highlights divergence in strategies for monooxygenase activity among heme enzymes. IDOs contain histidine residues as their proximal heme ligand, which provides an interesting contrast with the cysteine-ligated P450s (Scheme 3A). An informative comparison is also provided by considering the recently discovered reactivity of heme-containing tryptophan hydroxylase (TPH) enzymes. Although not classified as part of the heme-dependent aromatic oxygenase family, TPH enzymes also contain histidine-ligated heme active sites and have binding pockets for l-Trp that are similar to those in IDO. But the products of the TPH reaction differ—TPH catalyzes a P450-like hydroxylation of tryptophan (Scheme 3B), most likely using a Compound I intermediate, but without the need for an axial Cys ligand as in P450.^43^ Evidence from other work demonstrates monooxygenase activity from histidine-ligated heme enzymes in the HDAO family, including MarE (which forms 2-oxoindole)^44,45^ and PrnB (which forms monodechloroaminopyrrolnitrin)^46^ (Scheme 3B). Notably, similar HPIC species have been identified in TDO.^47,48^ Like MarE, IDO has also been shown^49^ to catalyze the formation of 2-oxoindole, as well as other mono- and dioxygenated indole products, albeit using peroxide, not O_2_. In this regard, there are emerging ideas^9^ that previous classifications of reactivity based on heme ligation are becoming blurred as we learn more about the heme-dependent aromatic oxygenase family of enzymes and that variables beyond heme ligation need to be considered when evaluating the determinants of heme reactivity.

In HeLa cells that have been induced with interferon-γ to upregulate IDO, we also identified the formation of both NFK *and* the cyclic HPIC product. This demonstrates that the bifurcated pathway shown in Scheme 3C is operational in live cells. Further evidence that the formation of HPIC occurs *in vivo* comes from the identification^50^ of HPIC in mammalian organs (such as kidney and lung sites) that are known to contain IDO.^51,52^ In addition, a closely related tricyclic hydroperoxide product is suggested as a possible regulator of vascular tone and blood pressure during inflammation.^53^ Together, this information suggests that IDO-catalyzed formation of HPIC, alongside the formation of NFK, is relevant *in vivo*.

## Conclusion

Alongside the known tryptophan dioxygenase activity of human IDO, leading to the formation of NFK, this study demonstrates that human IDO also has monooxygenase activity, leading to the formation of a cyclic HPIC product *in vitro* and in cells. The balance of dioxygenase/monooxygenase activity varies between different substrates. In the case of S-l-Trp—a known inhibitor of IDO—exclusive formation of the cyclic HPIC product is observed. For the methylated derivative of l-Trp (1-Me-l-Trp), which was once considered an inhibitor but is now known as a slow substrate,^26^ and for 5-OH-Trp, no cyclic product is observed. There is evidence that this bifunctional monooxygenase/dioxygenase behavior and formation of cyclic species could be quite general in the heme-dependent aromatic oxygenase family,^47,48^ including in the PrnB enzyme,^46^ which also forms a cyclic species.^28,54,55^ This offers a different perspective on the long-standing need to control tryptophan levels in cells. IDO is an important therapeutic target because many types of cancer cells overexpress either IDO or TDO, or both, as a mechanism to evade immune destruction.^56^ Redirecting enzyme activity toward monooxygenase, rather than dioxygenase, activity would deplete l-Trp levels without increasing NFK concentrations at the same time, which has not been previously considered.^56^ This may be helpful in unraveling the complicated interplays^54^ that connect tryptophan and kynurenine pathway metabolites in cells.

## Supplementary Material



## Notes

Details of materials and methods, computational methods, Supplementary Figures S1–S15, and Supplementary Tables S1–S4 (PDF)
