# Correction to “Monooxygenase Activity of Indoleamine 2,3-Dioxygenase”

**DOI:** 10.1021/jacs.6c08337

**Published:** 2026-06-26

**Authors:** Ali B. Lubis, Anna J. Bailey, Marko Hanževački, Christopher Williams, Mehul Jesani, Lola González-Sánchez, Christopher J. Arthur, Hannah C. Wilson, Andrea E. Gallio, Peter C. E. Moody, Matthew P. Crump, Adrian J. Mulholland, Allen M. Orville, Jonathan Clayden, Emma L. Raven

**Affiliations:** † School of Chemistry, Cantock’s Close, 1980 University of Bristol, Bristol BS8 1TS, U.K.; ‡ Research Complex at Harwell, 120796 Harwell Science and Innovation Campus Didcot, Didcot, Oxfordshire OX11 OFA, U.K.; § Centre for Computational Chemistry, School of Chemistry, Cantock’s Close, University of Bristol, Bristol BS8 1TS, U.K.; ∥ Institute for Structural and Chemical Biology, Department Molecular and Cell Biology, 4488 University of Leicester, Leicester LE1 7RH, U.K.; ⊥ Department of Physical Chemistry, University of Salamanca, Salamanca 37008, Spain; # Diamond House, Harwell Science and Innovation Campus, Diamond Light Source Ltd., Didcot, Oxfordshire OX11 0DE, United Kingdom

In the version of the article initially published, Allen Orville and Anna Bailey should have been listed with an additional affiliation as Diamond Light Source Ltd., Diamond House, Harwell Science and Innovation Campus, Didcot, Oxfordshire, OX11 0DE, United Kingdom.

In the original paper, Gonzáles-Sánchez has two affiliations. Only one affiliation should have been listed: Department of Physical Chemistry, University of Salamanca, Salamanca 37008, Spain.

The corrected affiliations are provided in the Author Information of this Correction.

Also, the statement in section 2.4 of the Supporting Information should have said “Reactions were initiated in a 500 mL volume by the addition of enzyme and were incubated overnight, *in the dark with tubes wrapped in aluminum foil,* to completion of enzymatic turnover”, with the modification to the original statement shown in italics. This has now been included in the updated Supporting Information file.

These changes do not affect the conclusion of the manuscript.

## Supplementary Material



## Notes

Details of materials and methods, computational methods, Supplementary Figures S1–S17, and Supplementary Tables S1–S4 (PDF)

