# Supplementary material for: Correction to “Monooxygenase Activity of Indoleamine 2,3-Dioxygenase”
Source: J Am Chem Soc. 2026 Jun 26;148(26):27985–6. doi: 10.1021/jacs.6c08337 (PMC13352586; doi:10.1021/jacs.6c08337)
Supplement: Supplementary file 1 [file ja6c08337_si_001.pdf]

Supporting Information

Monooxygenase Activity of Indoleamine 2,3-Dioxygenase

Ali B. Lubis[1], Anna J. Bailey[1,2], Marko Hanževački[3], Christopher Williams[1], Mehul Jesani[1], Lola González-Sánchez[4], Christopher J. Arthur[1], Hannah C. Wilson[1], Andrea E. Gallio[1], Peter C. E. Moody[4], Matthew P. Crump[1], Adrian J. Mulholland[3], Allen M. Orville[2], Jonathan Clayden[1], and Emma L. Raven[1,*]

[1] School of Chemistry, University of Bristol, Cantock's Close, Bristol, BS8 1TS, United Kingdom
[2] Research Complex at Harwell, Harwell Science and Innovation Campus, Didcot, Oxfordshire, Didcot OX11 ODE, United Kingdom
[3] Centre for Computational Chemistry, School of Chemistry, Cantock's Close, University of Bristol, Bristol, BS8 1TS, UK
[4] Department of Physical Chemistry, University of Salamanca, Salamanca, 37008, Spain
[5]Institute for Structural and Chemical Biology, Department Molecular and Cell Biology, University of Leicester, Leicester, LE1 7RH, United Kingdom

TABLE OF CONTENTS

1. GENERAL INFORMATION S2
   1.1 Materials S2
   1.2 Instrumentation S2
2. EXPERIMENTAL PROCEDURES S2
   2.1 Protein Expression and Purification S2
   2.2 Synthesis of NFK S2
   2.3 Synthesis of HPIC S2
   2.4 Enzymatic Assays S3
   2.5 Cell Culture S3
   2.6 Immunoblotting S3
   2.7 Computational methods S3
   2.7.1 System Preparation S4
   2.7.2 Molecular Dynamics Simulations S4
   2.7.3 Gas Phase DFT Calculations S4
3. FIGURES S6
4. TABLES S23
5. REFERENCES S24

1. GENERAL INFORMATION

1.1 Materials

Chemicals and reagents were procured from commercial sources and used without further purification. Acetic anhydride acid, formic acid, tetrahydrofuran, ethanol, water, L-tryptophan, Rose Bengal, dimethyl sulfide, acetic acid, methanol, ascorbic acid, methylene blue, catalase, disodium phosphate, sodium chloride, 1-Me-L-Trp, L-tryptophanol, and 5-OH-L-tryptophan were obtained from Sigma-Aldrich Chemical Co. Monosodium phosphate was obtained from Melford Biolaboratories Ltd. Tris base was obtained from Fisher Bioreagents. β-[3-benzo(b)thienyl]- L-alanine (S-L-Trp), L-tryptophan (indole-ring-2 ^{13}C), and fully-labelled-^{13}C L-tryptophan were obtained from Cambridge Isotope Laboratories, Inc. α-MEM medium and fetal bovine serum were obtained from Gibco. Interferon-γ (IFN-γ) was obtained from PeproTech.

1.2 Instrumentation

UV-visible spectra were recorded using either Perkin-Elmer Lambda 40 or Agilent 8453 (1 mm slit width) UV-visible spectrophotometers at 298 K using a 0.5 ml or 1 ml quartz cuvette. Baseline corrections were made against the buffers or solvents used in analyses.

LC-MS data were acquired using a Thermo Orbitrap Elite (Thermo Scientific) spectrometer, equipped with an electrospray ionisation (ESI) source and integrated with an Ultimate 3000 HPLC system. Analyses were performed in positive ion mode using a C18 column and a solvent mixture of 0.1% acetonitrile in H_2O, in ratios of 2:8 or 1:9, with measurements conducted at 298 K.

NMR spectra were recorded using a Jeol ECS 400 spectrometer fitted with an autotune probe, a Varian 600 Cryo spectrometer fitted with a cryo-enhanced triple resonance probe and a Bruker Avance III HD 700 fitted with a 1.7 mm cryo-enhanced micro coil probe. All measurements were conducted at 298 K. Chemical shifts (δ) are reported in parts per million (ppm) and are referenced to the D_2O solvent peak (δ H: 4.79 ppm).

2. EXPERIMENTAL PROCEDURES

2.1 Protein Expression and Purification

Human indoleamine 2,3-dioxygenase (hIDO) was expressed in *E. coli* and purified as described previously.[1] Protein concentrations were determined using the absorption coefficient for hIDO of $\varepsilon_{404} = 172$ mM^{-1}cm^{-1}.[2]

2.2 Synthesis of *N*-Formylkynurenine (NFK)

The initial reactants were prepared by mixing 0.089 ml (0.93 mmol) of acetic anhydride and 0.043 ml (1.15 mmol) of formic acid, at 0 °C. Subsequently, the temperature of the solution was increased to 60 °C before 0.075 g (0.36 mmol) L-kynurenine, and 0.156 ml of tetrahydrofuran were added and stirred overnight at room temperature. The solution was then placed in an ice bath and diethyl ether (2.00 ml) was added to precipitate the product which was obtained by filtration. The NFK product was characterised by LC-MS, ^{1}H-NMR and HSQC (Figure S1).

2.3 Synthesis of 3a-hydroxy-1,2,3,3a,8,8a-hexahydropyrrolo[2,3-b]indole-2-carboxylic acid (HPIC)

The reaction solution was prepared by mixing 300 ml of ethanol/water (5% v/v) and 15 mg (0.02 mmol) of Rose Bengal. L-tryptophan (1.02 g, 5 mmol) was then added, followed by bubbling of the solution with O_2. During the reaction, the mixture was irradiated at 554 nm using a LED lamp for 5 hours at room temperature. After this time, 4.23 g (68 mmol) of dimethyl sulfide was added and the solution was stirred for an hour until the starch-KI test became negative. The mixture then was acidified with 3 ml of acetic acid and evaporated before being applied to an HPLC column (C18 column, using methanol/water (1:9) as solvent) to attain the product. The HPIC product contains two chiral carbons, which makes it possible to separate *cis*- and *trans*-HPIC using a standard C18 column as they elute separately (Figure 1A(i)). The spectrum

of the HPIC product shows maxima at 237 and 292 nm and was characterised using LC-MS, ^{1}H-NMR and HSQC (Figures 1A(i) and S3) and mass spectrometry. ^{1}H-NMR and HSQC spectra of the product were consistent with previous reports.

2.4 Enzymatic Assays

Enzymatic assays using hIDO with a range of substrates (L-Trp, L-tryptophanol, 1-Me-L-Trp, 5-OH-L-Trp and S-L-Trp, Scheme 2A) were carried out in 50 mM Tris-HCl buffer (pH 8.0) according to previous methods[3] and consisted of ascorbic acid (20 mM), enzyme (2.5 µM), methylene blue (1 µM), catalase (1 µM) and L-tryptophan (1 mM). Reactions were initiated in a 500 µl volume by the addition of enzyme and were incubated overnight, in the dark with the tubes wrapped in aluminium foil, to completion of enzymatic turnover. Solutions were used directly for further analyses in LC-MS and NMR. Formation of NFK was monitored at 321 nm; formation of HPIC is not visible by UV-visible spectrophotometry at 292 nm, and is only observed by LC-MS and NMR. Trace amounts of HPIC are observed in the absence of ascorbate as reductant, but the enzymatic turnover rate is very low in the absence of ascorbate. Higher concentrations of ascorbate gave marginally higher concentrations (1.3-fold increase from 10 µM to 20 µM ascorbate. However further increase in ascorbate concentration does not give any significant change in HPIC formation. Using NADH (10, 20, 100 and 250 µM) as reductant we also observed HPIC formation under enzymatic assay conditions. Product ratios are given in Table 1 of the main text, and were obtained by integration of LC-MS peaks.

2.5 Cell Culture

HeLa cells were cultured overnight in a 96-well culture plate in growth medium (α-MEM, Gibco) supplemented with non-essential amino acids (αα) and 10% fetal bovine serum. Note that the α-MEM media used for cell culture contains 0.28 mM ascorbic acid, which is much lower than in the *in vitro* assays (20 mM) as in section 2.4. Following incubation, interferon-γ (IFN-γ) (5 ng/mL) and L-tryptophan (either 0 or 400 µM) were added into the cultures. The cells were then incubated at 37 °C in 5% CO_2 for 24 hours. After incubation, the culture medium was collected into microtubes and analyzed directly by LC-MS without further purification.

2.6 Immunoblotting

Cells were lysed using radio-immuno precipitation assay (RIPA). Protein concentration was measured using a Pierce BCA (bicinchoninic acid) assay (ThermoFisher). Protein lysate (7 µg) was prepared to a final volume of ≤ 10 µL using 4 x Laemmli buffer before boiling. Boiled samples were loaded into a 17-well Nu-PAGE 4-12 % BisTris gel (11.0 mm x 17 well; Invitrogen). Electrophoresis was carried out at 160 V for 1 hour. The gel was transferred onto a low-fluorescence PVDF membrane (pore size 0.2 µM; Thermo Scientific 22860) using the Xcell II Blot module (Invitrogen) for a wet transfer at 30 V for 70 minutes. The membrane was blocked with blocking buffer (5 % Blotto dry milk dissolved in Tris Buffered Saline-Tween (TBST) containing 0.1 % Tween) for 1 hour at room temperature. hIDO was probed with an overnight incubation at 4 ˚C with rabbit anti-hIDO (antibodies.com A8946, dilution 1:1000). β-actin was used as an internal control, probed using mouse anti-actin (antibodies.com A85272, dilution 1:2000). After primary incubation, membranes were washed with TBST (3 x 5-minute washes) on a rocking shaker, followed by a 1-hour incubation at room temperature with goat anti-mouse secondary antibody (Biotim 0065, dilution 1:20,000) and donkey anti-rabbit secondary antibody (Biotim 20344, dilution 1:20,000). Membranes were washed again with TBST (3 x 5 minutes) on a rocking shaker before imaging using a Licor Odyssey Fc imager with 2 min acquisition on the 700 and 800 channels. Images were analysed using Empiria Studio® Software.

2.7 Computational methods

2.7.1 System Preparation. To model the enzyme-substrate complex, we utilized the crystal structure of human indoleamine 2,3-dioxygenase (hIDO) bound to its natural substrate L-tryptophan (L-Trp) and heme (PDB ID: 5WMU).[4] Water molecules observed in the crystal structure were retained, and the cyanide molecule coordinating the iron atom of the heme was replaced with an oxygen molecule. The missing loop (residues 363-373) was modeled using the SWISS-MODEL homology modeling server.[5] Protonation states of titratable residues were determined using the H++ server.[6] The AMBER ff19SB force field was employed to describe standard protein residues.[7] Zwitterionic L-Trp substrate parameters were obtained from the AMBER parameters database.[8] The 3-(1-benzothiophen-3-yl)-L-alanine (S-L-Trp) substrate structure was extracted from a standalone ligand database (PDB ID: 4OG), with hydrogen atoms added to its zwitterionic form. Atom types were then described using the general AMBER force field (GAFF)[9], and partial charges were calculated using the AM1-BCC method.[10] S-L-Trp was manually docked into the hIDO active site in a conformation similar to L-Trp. A similar approach was employed to investigate binding of 1-Me-L-Trp and 5-OH-Trp to hIDO. The force field parameters for the O_2-bound heme were developed using the standard metal center parameter builder (MCPB) protocol.[11, 12] Each system was solvated in a truncated octahedron box of TIP3P water molecules,[13] ensuring a minimum distance of 10 Å between the solute and the water box edge. Neutralization was achieved by adding Na^+ counterions.[14]

2.7.2 Molecular Dynamics Simulations. Initial unrestrained energy minimization was conducted in two sequential phases, commencing with 1000 steps utilizing the steepest descent method, followed by 9000 steps employing the conjugate gradient algorithm. The system temperature was gradually increased from 100 K to 298 K over 1 ns using the NVT ensemble at constant volume. A subsequent 1 ns restrained relaxation at 298 K was performed under a constant pressure of 1 bar, with positional harmonic restraints applied to all solute atoms using a force constant of 10 kcal mol^{-1} $Å^{-2}$. Three consecutive 1 ns relaxation simulations were executed at constant pressure, maintaining positional restraints on the protein backbone heavy atoms. Throughout these simulations, the positional restraint force constant was progressively decreased from 10 to 0.1 kcal mol^{-1} $Å^{-2}$, facilitating gradual system equilibration and structural refinement. The unrestrained production simulations were performed for a minimum duration of 400 ns using the NPT ensemble at 298 K and 1 bar pressure. Each replica was repeated three times, giving a total simulation time of 1.2 μs per system. The simulations were configured with a 1 fs time step and an 8 Å cutoff for non-bonded interactions. Temperature control was achieved through a Langevin thermostat[7] with a collision frequency of 1 ps^{-1}, while pressure was regulated using an isotropic Monte Carlo barostat.[15] The SHAKE algorithm was systematically applied to constrain bonds involving hydrogen atoms.[16] All MD simulations were executed using the *pmemd.cuda* module of the AMBER22 software package.[17] Comprehensive trajectory analysis was conducted utilizing the *cpptraj* module, with structural visualization accomplished through PyMOL 3.1.3.1.[18]

2.7.3 Gas Phase DFT Calculations. To elucidate the experimentally observed products and optimize the structures of intermediates in proposed mechanisms, we conducted comprehensive computational analyses of turnover of both L-Trp and S-L-Trp. Our investigation encompassed monooxygenated (epoxide and HPIC) and dioxygenated (dioxetane and NFK) systems. Additionally, our calculations included the hypothetical conversion of 1-Me-L-Trp and 5-OH-Trp to cyclic HPIC products. The production of 6-membered ATPI and 5-membered HPIC analogue products obtained after the monooxygenation of L-tryptophanol was also studied. We performed preliminary geometry optimizations using the Gaussian16 software package in the gas phase.[19] The calculations employed the B3LYP/6-31G(d) level of theory with Grimme's D3 dispersion correction and Becke-Johnson damping (D3BJ). Utilizing the optimized geometries as starting points, we conducted extensive conformational sampling using Grimme's CREST 2.12 software package. [20, 21] The conformer generation process employed the semiempirical GFN2-xTB method, as implemented in the xTB 6.6.1 program,[22]

for initial geometry optimizations and conformer generation. We extracted all conformers within a 1 kcal mol^{-1} energy window from the lowest energy structure. These selected conformers underwent further optimization in the gas phase using the B3LYP-D3BJ/6-31G(d) level of theory. To obtain more accurate electronic energies, we performed single-point calculations on the optimized structures using the B3LYP-D3BJ/def2-TZVP level. The non-covalent interactions (NCIs) were characterized using Multiwfn software calculating the reduced density gradient (RDG).[23] The VMD 1.9.3[24] was used to generate and visualize isosurface maps of weak interactions.

To further clarify the electronic effects associated with N-methyl substitution, we performed DFT calculations comparing L-Trp and 1-Me-L-Trp. The analyses included natural bond orbital (NBO) charges,[25, 26] electrostatic potential (ESP) distributions, and HOMO-LUMO energy gaps at the B3LYP-D3BJ/def2-TZVP//B3LYP-D3BJ/6-31G(d) level of theory. The computed HOMO-LUMO energy difference for L-Trp (117.1 kcal/mol) was slightly larger than that for 1-Me-L-Trp (114.4 kcal/mol), suggesting a marginally higher stability and hence lower reactivity of l-Trp. Consistently, the dipole moment of 1-Me-L-Trp (4.80 D) was greater than that of L-Trp (4.56 D), supporting enhanced polarity and reactivity upon N-methylation. The ESP maps revealed distinct potential values near the indole nitrogen region (0.06 for N-H in L-Trp versus 0.02 for N-Me in 1-Me-L-Trp), consistent with reduced charge delocalization from nitrogen toward the adjacent C2 carbon in the N-methylated derivative (-0.016) compared to L-Trp (-0.024). This interpretation aligns with the NBO analysis showing a less negative charge on N1 in 1-Me-L-Trp (-0.321) compared to L-Trp (-0.499). The higher electron-donating capacity of N1 in L-Trp facilitates electron transfer to the C2 position, which plays a critical role in HPIC formation.

2.7.4 Protein-ligand docking calculations. Protein-ligand docking calculations were performed using the GOLD 2022.3 software package.[27] The representative protein structure of IDO was obtained from MD simulations of the enzyme in complex with the heme cofactor and L-Trp. Before docking, the bound L-Trp substrate, the dioxygen molecule coordinated to the heme iron, and all water molecules were removed. The binding site was defined as the region encompassing the heme cofactor and all protein residues having at least one atom within 10 Å of the bound L-Trp in the reference structure. Docking was carried out employing the ChemPLP fitness function to evaluate and rank the predicted poses.[28]

3. FIGURES

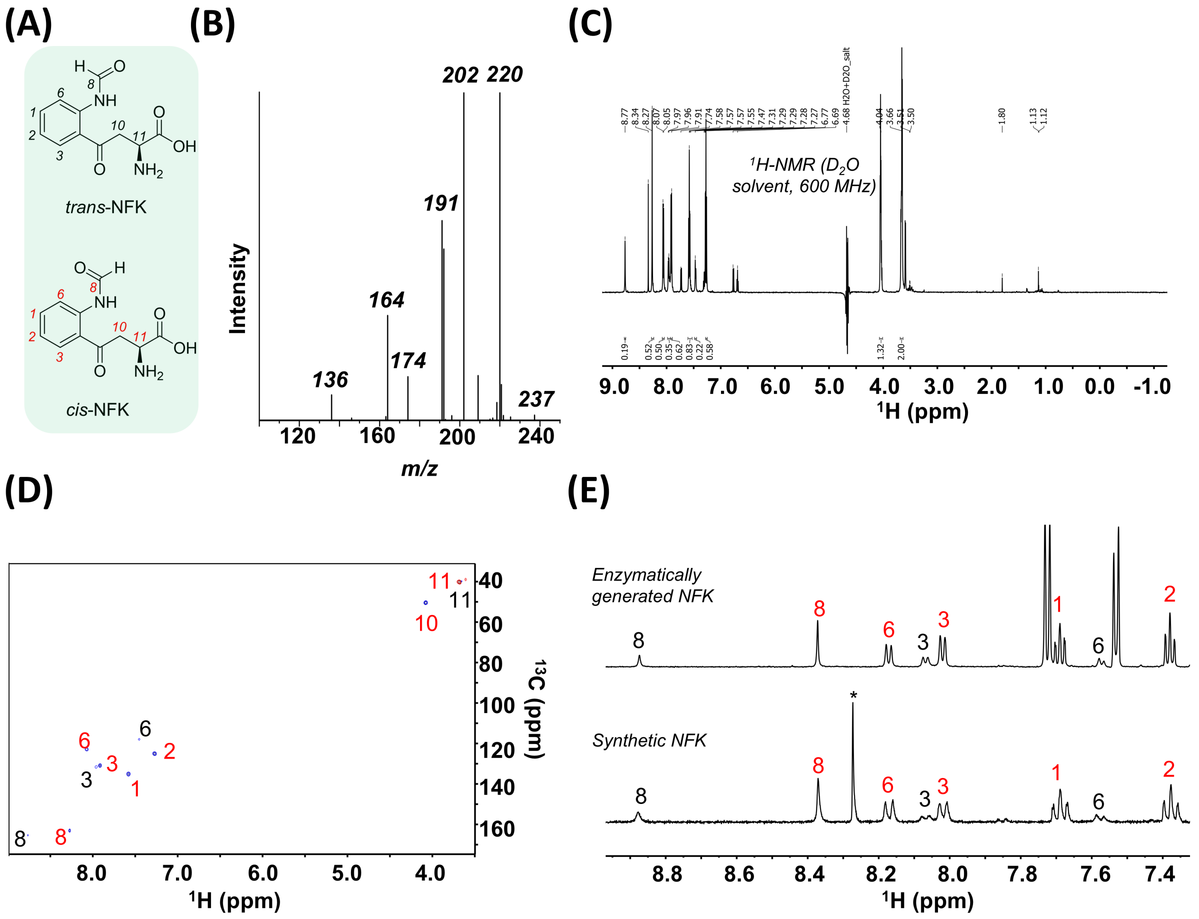

Figure S1. **Synthesis of NFK**. **(A)** Chemical structures of *trans*- and *cis*-NFK. The synthesized NFK (see Methods) was characterized by **(B)** mass spectrometry, **(C)** ^{1}H-NMR, and **(D)** HSQC spectra. In **(D)** signals relating to individual carbon atoms for *trans*-NFK (in black) and *cis*-NFK (in red) are indicated and labelled according **(A)**. **(E)** Comparative ^{1}H-NMR spectra for the synthetic NFK (bottom spectrum) and NFK generated enzymatically in the hIDO assay (top spectrum). The peak labelled * is residual formic acid (used in the synthesis of NFK). The ^{1}H-NMR spectrum identifies peaks for both *trans*- (in black) and *cis*- (in red) isomers, in **(A)**, arising from the free rotation of the formyl group. Detailed NMR data are provided in Table S2. PubChem Compound Identifier (CID) number for NFK: 910. Note that *trans*- and *cis*-NFK are rotamers not geometric isomers, so they have different signals in ^{1}H or ^{13}C NMR, but are not separable stereoisomers).

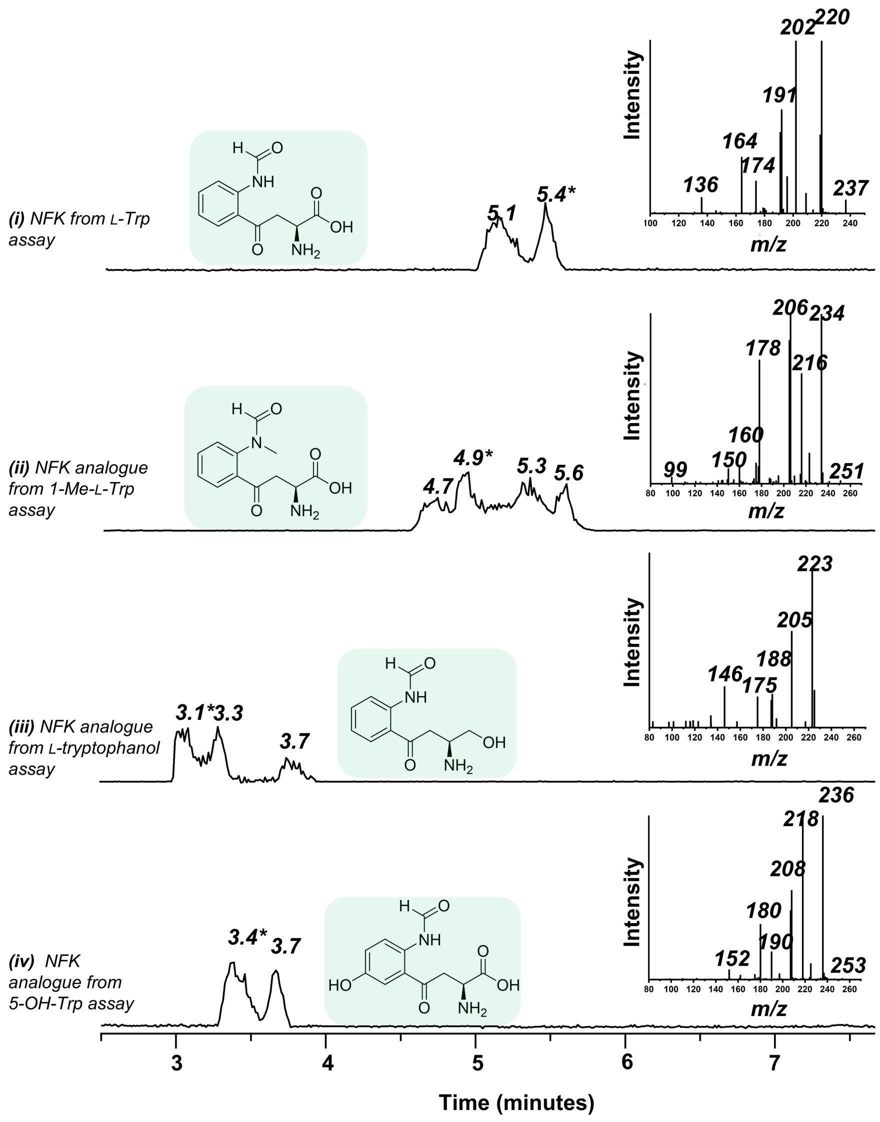

Figure S2. **Enzymatic formation of NFK and NFK analogues by hIDO.** LC-MS analyses showing the formation of NFK and NFK analogues derived on reaction of **(i)** L-Trp, **(ii)** 1-Me-L-Trp, **(iii)** L-tryptophanol and **(iv)** 5-OH-Trp with hIDO. The inset in **(i) – (iv)** shows the mass spectrum of NFK identified from the peak labelled with a * in each case. All the peaks in each of the LC analyses gave the same fragmentation pattern in the MS analyses as the peak labelled *. Expected fragmentation patterns for each of the products formed in **(i) – (iv)** are given in Figures S5-S7 and S9, respectively. Detailed accurate mass data are provided in Table S3.

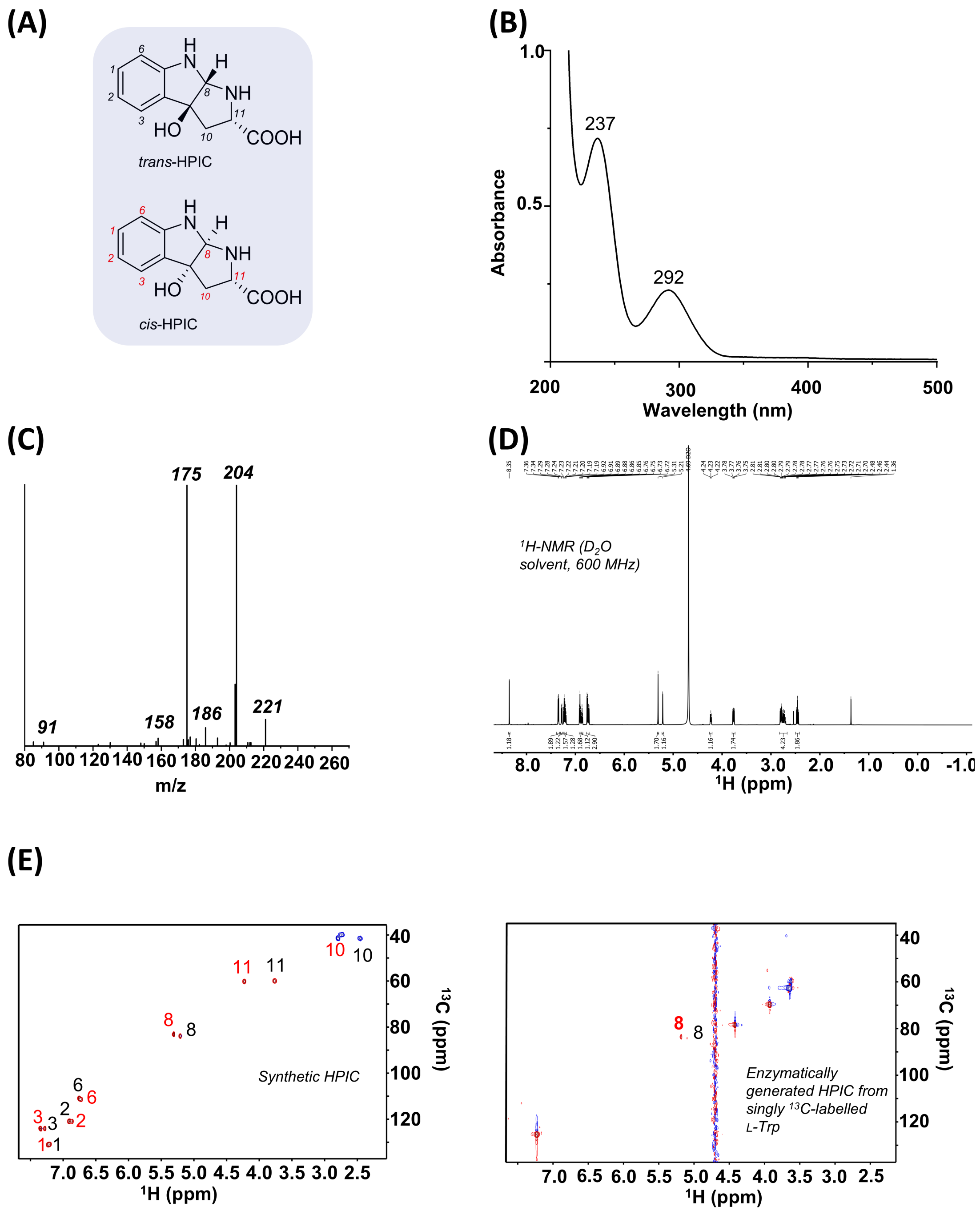

Figure S3. **Synthesis of HPIC.** **(A)** Chemical structures of *trans*- and *cis*-HPIC. The synthesized HPIC (see Methods) was characterized by **(B)** UV-visible spectra, **(C)** mass spectrometry, and **(D)** ^{1}H-NMR. **(E)** Comparative HSQC spectra highlighting C8 of both *trans*-HPIC (black) and *cis*-HPIC (red), observed at 5.0 – 5.5 ppm (^{1}H) and approximately 80 ppm (^{13}C), are shown for the synthetic HPIC (left) and for HPIC enzymatically generated in the hIDO assay with singly ^{13}C-labelled L-Trp (right) (see Figure 1). In the synthetic HPIC spectrum (left), signals relating to individual carbon atoms for *trans*-HPIC (in black) and *cis*-HPIC (in red) are indicated and labelled according **(A)**. Detailed NMR data are provided in Table S4. PubChem Compound Identifier (CID) number for HPIC: 10331043.

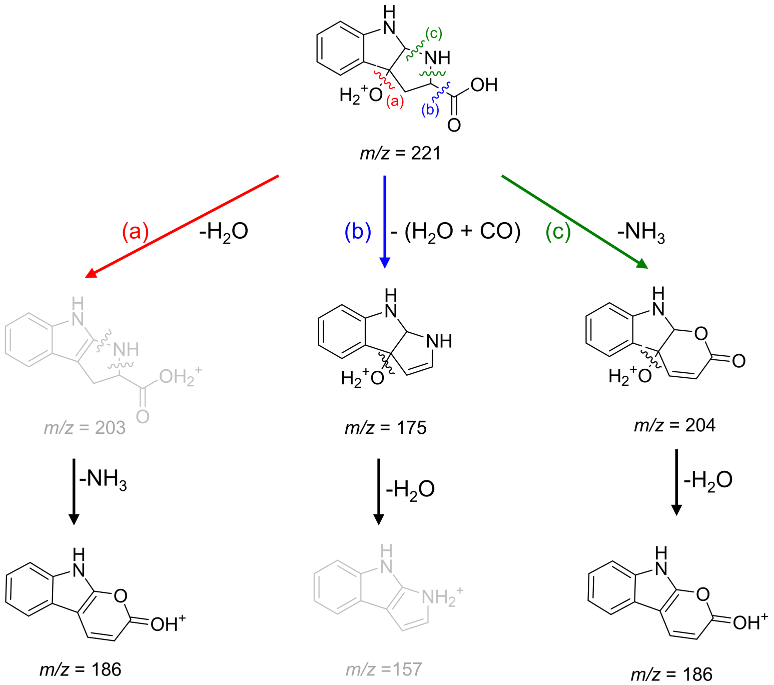

Figure S4. Predicted mass fragmentation pattern of HPIC, as identified in the positive ionization mode of the LC-MS experiment. *cis*-HPIC and *trans*-HPIC have the same fragmentation pattern. Protonated HPIC (*m/z* =221) fragments in parallel via steps (a), (b) and (c). Step (a) is release of H_2O; step (b) is release of CO + H_2O; step (c) is release of NH_3. All the species shown are observed in the mass spectrum of the *cis* isomer of HPIC, as shown in the inset in Figure 1A(i), with the exception of the species shown in grey (*m/z* = 203, 157), which are only observed in the inset in Figure 3B(ii),(iii).

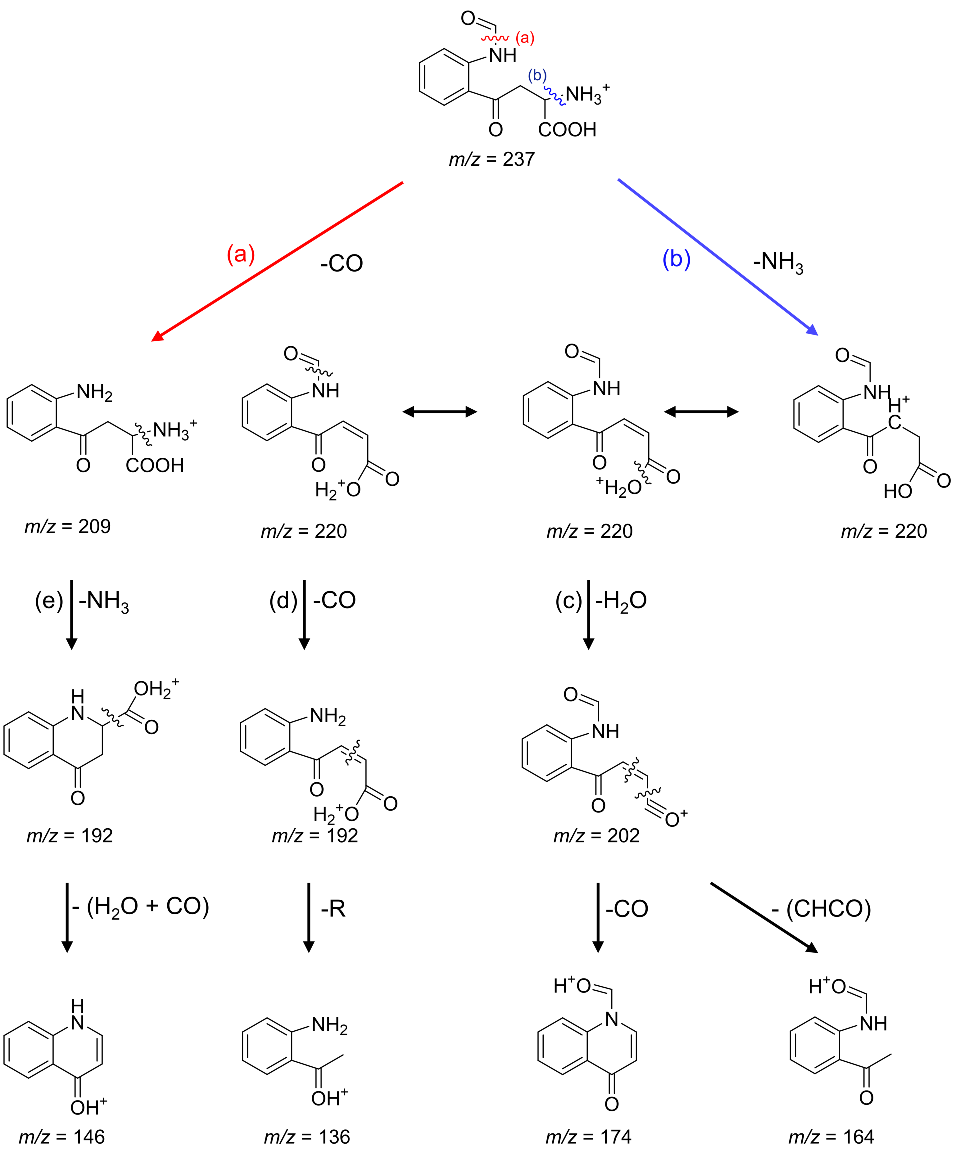

Figure S5. Predicted mass fragmentation pattern of NFK, as identified in the positive ionization mode of the LC-MS experiment. Protonated NFK (*m/z* = 237) fragments in parallel via steps (a) and (b). Step (a) is release of NH_3; step (b) is release of CO. All of the species identified above are observed in the mass spectra in Figures S2(i) and 3A(ii),(iii).

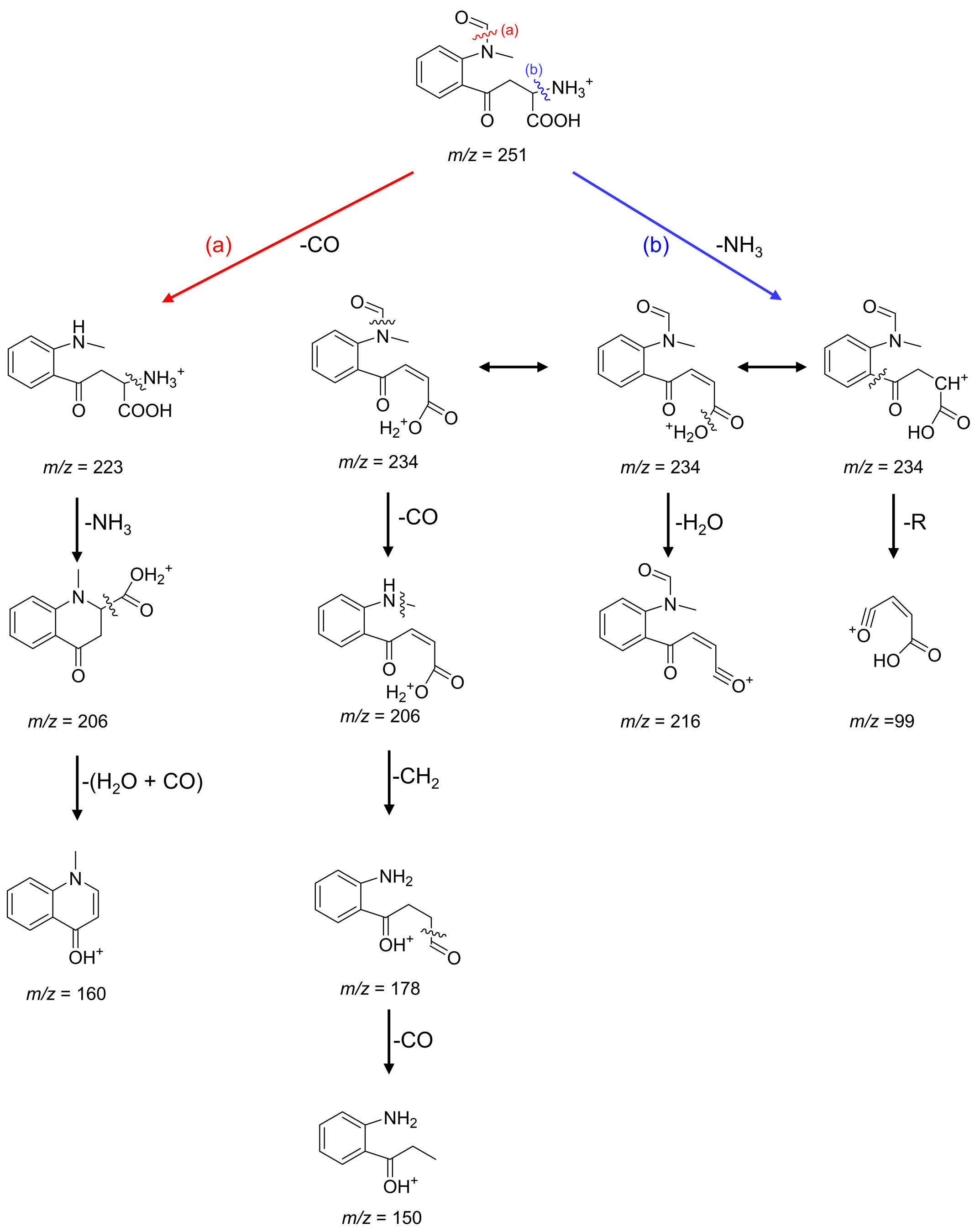

Figure S6. Predicted mass fragmentation of *N*-formyl-methylkynurenine (Me-NFK), as identified in the positive ion mode of the LC-MS experiment. The fragmentation pattern (steps (a) and (b)) is similar to that in Figure S5 for NFK. All the species identified above are observed in the mass spectra in Figure S2(ii).

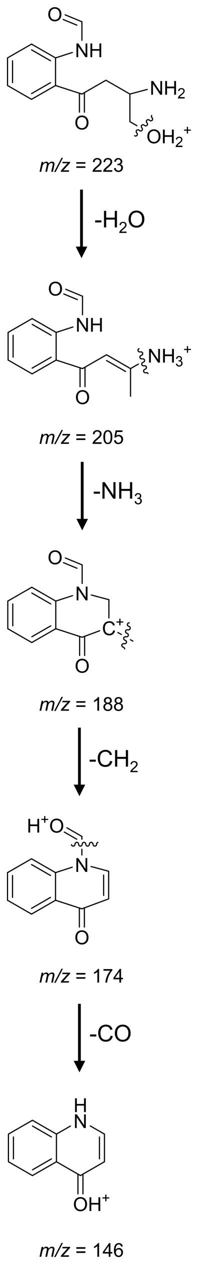

Figure S7. Predicted mass fragmentation of the NFK analogue derived from the reaction of L-tryptophanol with hIDO, as identified in the positive ionization mode of LC-MS. All the species identified above are observed in the mass spectrum in Figure S2(iii).

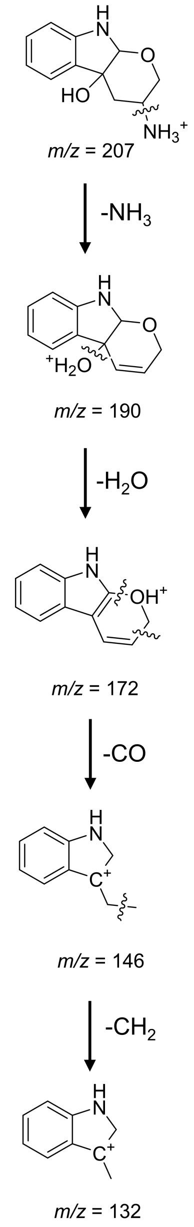

Figure S8. Predicted mass fragmentation pattern of the cyclic ATPI product, Scheme 2B(ii), derived from the reaction of L-tryptophanol with hIDO. All the species identified above are observed in the mass spectrum in Figure 1A(iii).

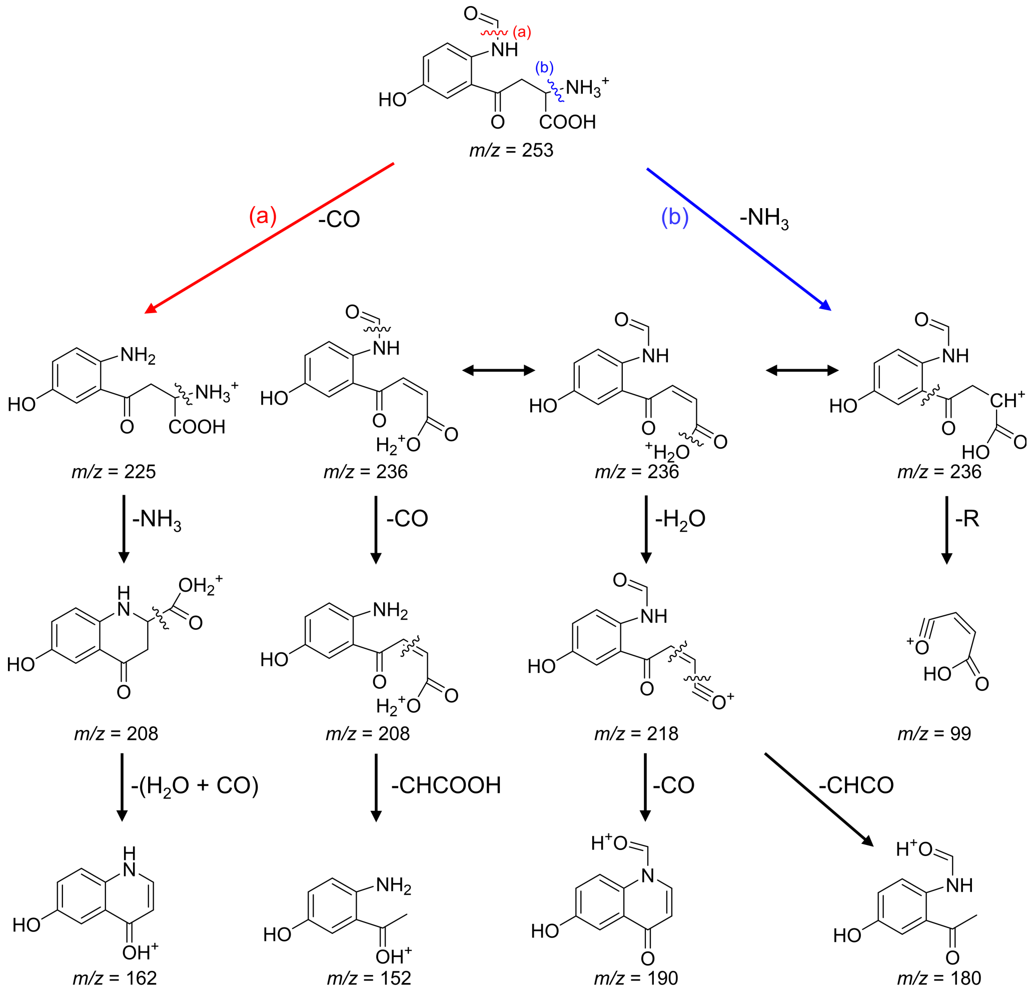

Figure S9. Predicted mass fragmentation of the NFK analogue derived from the reaction of 5-OH-Trp with hIDO, as identified in positive ionization mode of LC-MS. The fragmentation pattern (steps (a) and (b)) is similar to that in Figure S5 for NFK. All the species identified above are observed in the mass spectrum in Figure S2(iv).

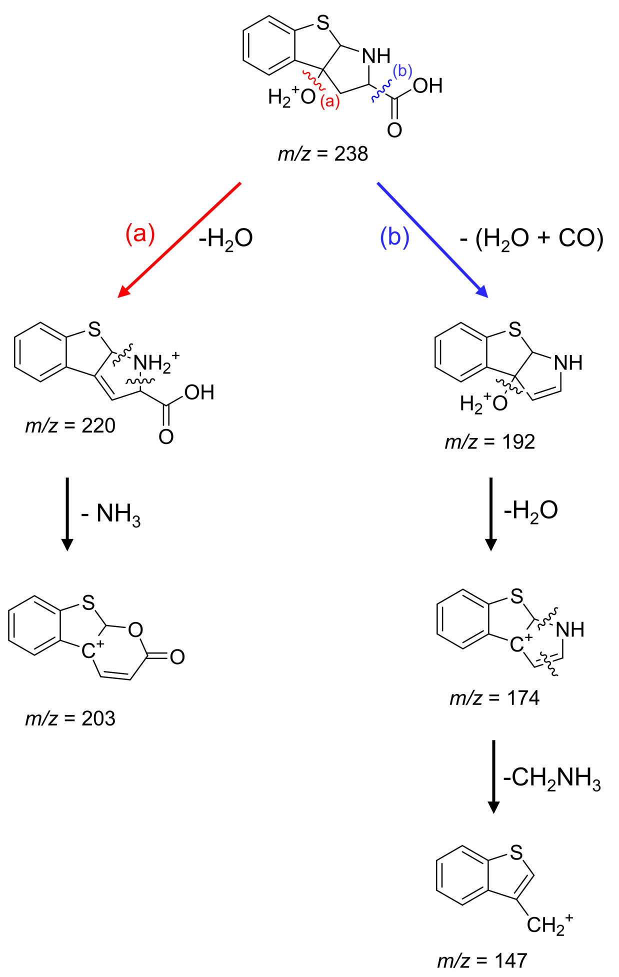

Figure S10. Predicted mass fragmentation of the HPIC analogue derived from the reaction of S-L-Trp with hIDO, as identified in the positive ionization mode of the LC-MS experiment. The protonated HPIC analogue (*m/z* =238) fragments in parallel via steps (a) and (b). All the species shown are observed in the mass spectrum of the *cis* isomer of HPIC analogue, as shown in the inset in Figure 1A(iv).

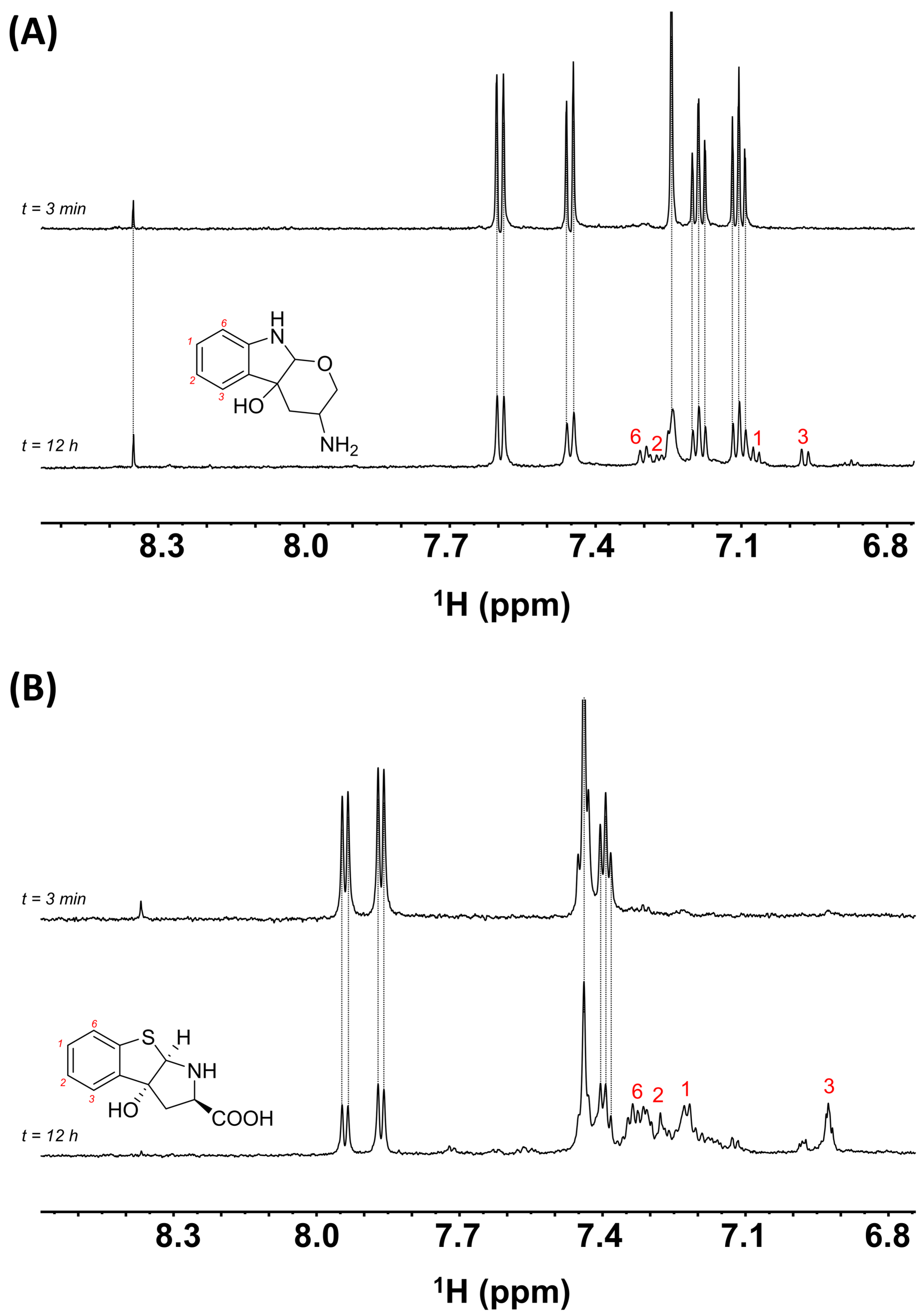

Figure S11. ^{1}H-NMR spectra showing the formation of cyclic products (Scheme 2B(ii),(iii)) in the hIDO-catalysed oxidation of **(A)** L-tryptophanol and **(B)** S-L-Trp. **(A)** At t = 3 minutes (top spectrum), only peaks associated with L-tryptophanol are observed; at t = 12 h (bottom spectrum), additional peaks (indicated in red) assigned to the aromatic protons of the cyclic ATPI product were observed. **(B)** At t = 3 minutes (top spectrum), only peaks associated with S-L-Trp are observed. At t = 12 h (bottom spectrum), additional peaks (indicated in red) assigned to the aromatic protons of the cyclic HPIC product were observed; these additional peaks are consistent with those of the synthetic HPIC (Figure S3D,E).

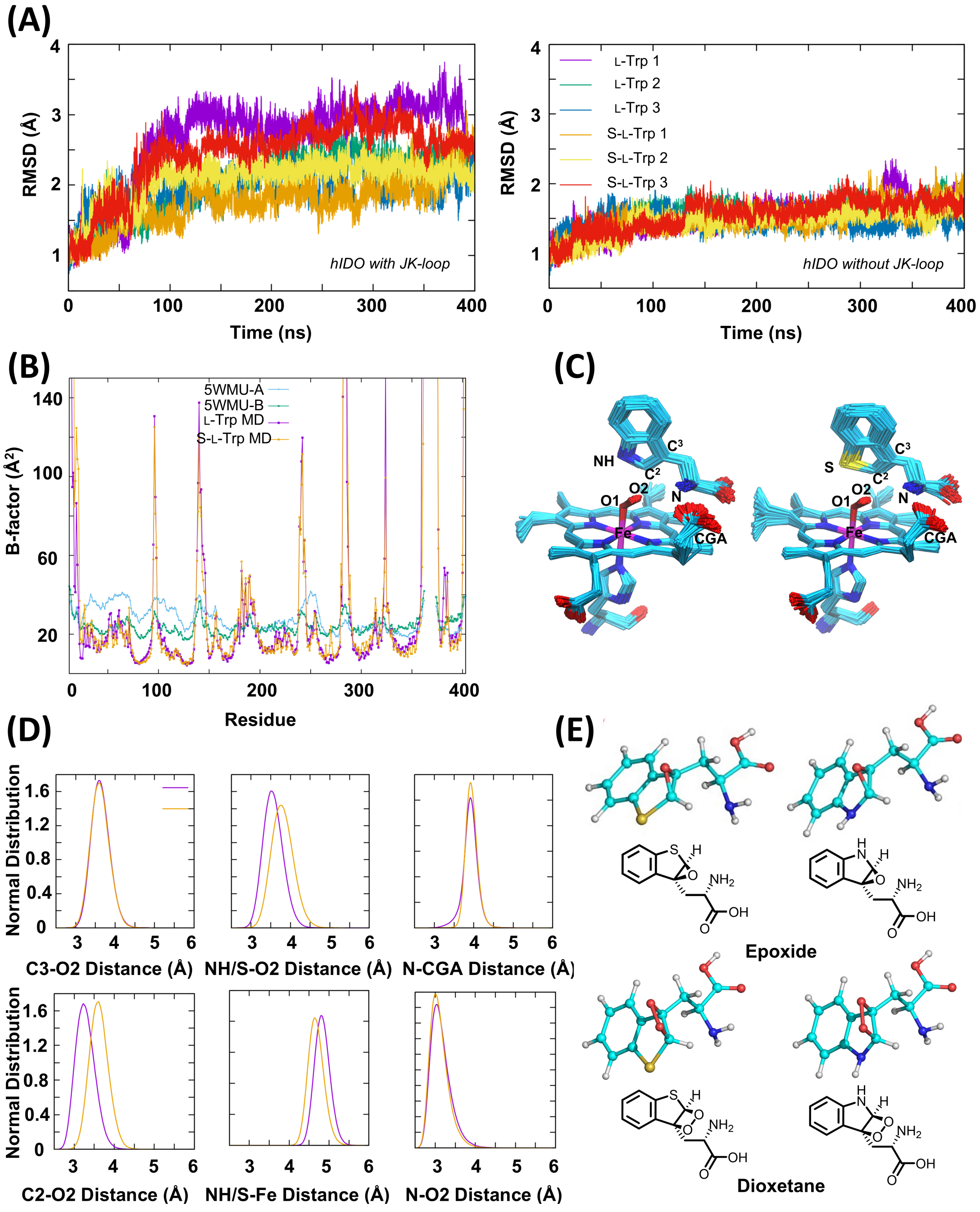

Figure S12. MD Simulations. **(A)** Root-mean-square deviation (RMSD) of protein backbone Cα atoms from L-Trp and S-L-Trp in complex with hIDO (left), and with hIDO lacking the loop region comprising residues 363-373 (JK-loop, right). Each plot represents an independent production simulation. **(B)** B-factors for Cα atoms, comparing experimental values taken from the PDB entry 5WMU (chain A and chain B) with results from MD simulations for L-Trp and S-L-Trp. These B-factor data support the RMSD analysis, indicating that the JK-loop exhibits a high flexibility and may contribute to substrate binding and product release. **(C)** Superimposed MD snapshots of L-Trp and S-L-Trp, highlighting key interactions with O_2 and heme. **(D)** Histograms of key distances between L-Trp and S-L-Trp with O_2 and heme. All atoms used for distance calculations are shown in **(C)**. **(E)** DFT structures of epoxide and dioxetane intermediates of L-Trp and S-L-Trp used in the relative energetics calculations.

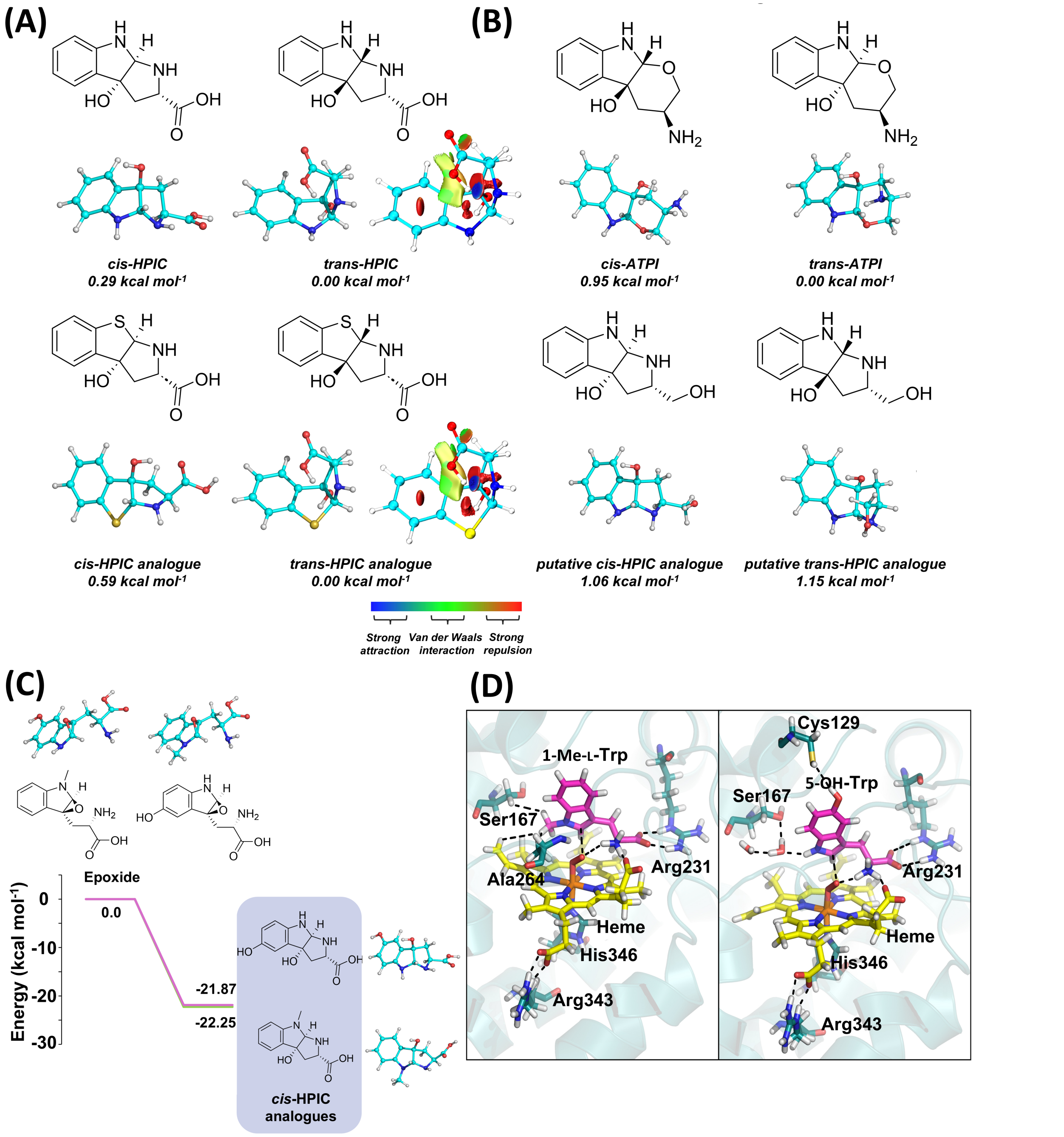

Figure S13. DFT and MD Calculations. **(A)** Lowest-energy conformers of *cis*- and *trans*-HPIC formed after the monooxygenation of L-Trp and S- L-Trp, with noncovalent interaction (NCI) analysis showing stabilizing intramolecular van der Waals interaction between carboxylic acid group and aryl ring. All calculations were carried out in vacuo at the B3LYP-D3BJ/def2-TZVP//B3LYP-D3BJ/6-31G(d) level of theory. **(B)** The lowest-energy conformers of *cis*- and *trans*-ATPI (featuring two six-membered rings and one five-membered ring), as well as those of the putative HPIC analogs (containing one six-membered ring and two five-membered rings) obtained after the monooxygenation of L-tryptophanol. **(C)** Relative energy diagram of *cis*-HPIC formation from the *cis*-epoxide intermediate in 1-Me-L-Trp and 5-OH-Trp. **(D)** Representative MD snapshots of hIDO in complex with 1-Me-L-Trp (left) and 5-OH-Trp (right) illustrate key interactions stabilizing the respective substrates. The conformation of 1-Me-L-Trp is constrained by enhanced hydrophobic contacts with Ser167, Ala264, and the heme cofactor, while 5-OH-Trp exhibits reduced flexibility due to a stabilizing hydrogen bond with Cys129. Collectively, these interactions limit the conformational plasticity required for cyclization, thus favoring formation of the experimentally observed NFK products over cyclic HPIC products.

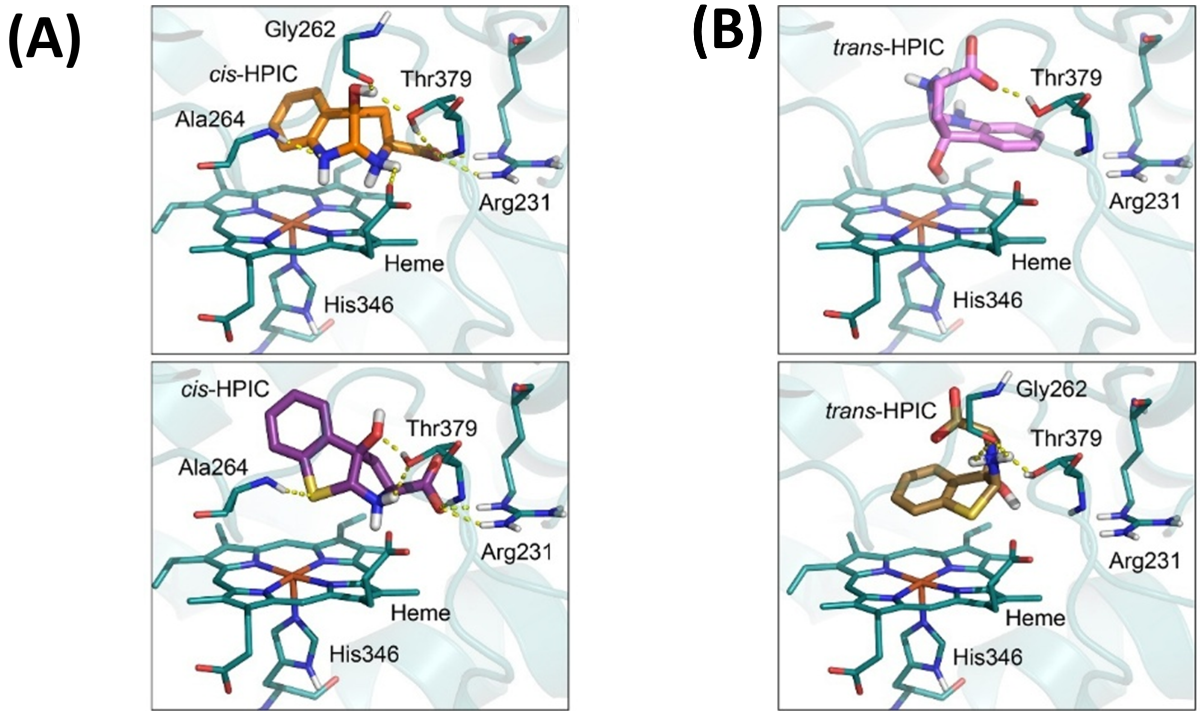

Figure S14. Structures of protein-ligand complexes illustrating key binding interactions. Best docking pose of **(A)** *cis*-HPIC and **(B)** *trans*-HPIC derived from L-Trp and S- L-Trp. Important hydrogen bonds are shown as yellow dashed lines. All nonpolar hydrogens are omitted for clarity. Details on the docking protocol and software are provided in the SI.

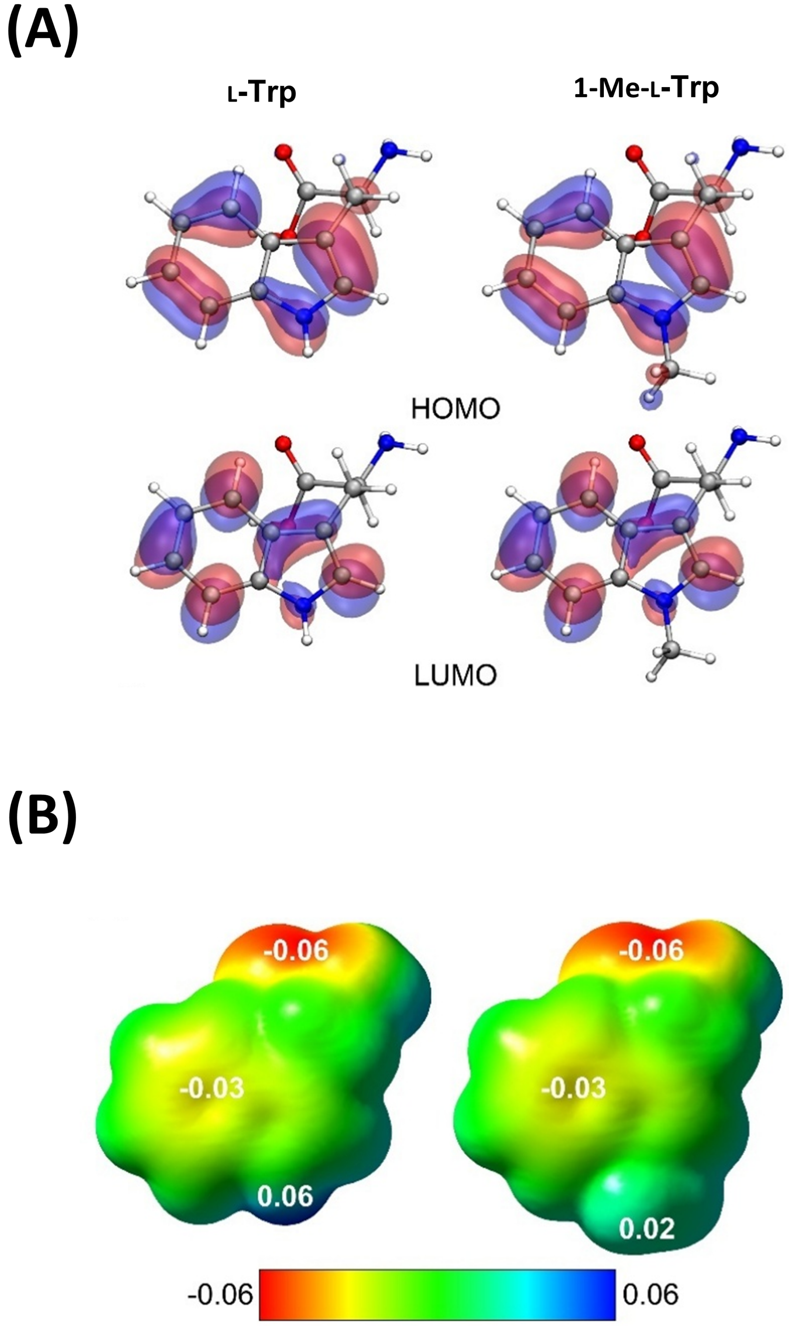

Figure S15. Electronic influence of *N*-methyl substitution on reactivity. (A) HOMO-LUMO isosurfaces and **(B)** electrostatic potential (ESP) maps of L-Trp and 1-Me-L-Trp computed at the B3LYP-D3BJ/def2-TZVP//B3LYP-D3BJ/6-31G(d) level of theory. HOMO and LUMO isosurfaces are displayed with an isovalue of 0.04; negative and positive phases are colored red and blue, respectively. ESP surfaces are mapped onto the total electron density with an isovalue of 0.0004.

(A)

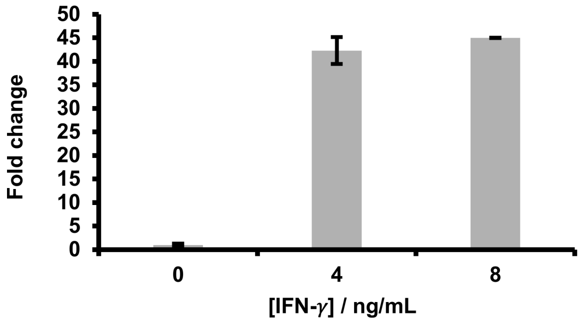

(B)

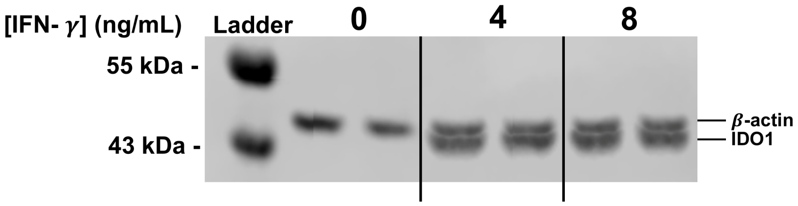

Figure S16. Immunoblot Analysis. (A) Quantitative immunoblotting of HeLa cells after 24-hour incubations with 0, 4 and 8 ng/mL IFN-γ, showing induction of hIDO expression. Cells not treated with IFN-γ (0 ng/mL) show no detectable hIDO expression. **(B)** Representative immunoblots corresponding to the quantitative analyses in **(A)**, using whole-cell lysates from HeLa cells treated under the same conditions. Membranes were imaged using a LI-COR Odyssey Fc system, and band intensities were quantified by densitometry and normalised to β-actin as a loading control.

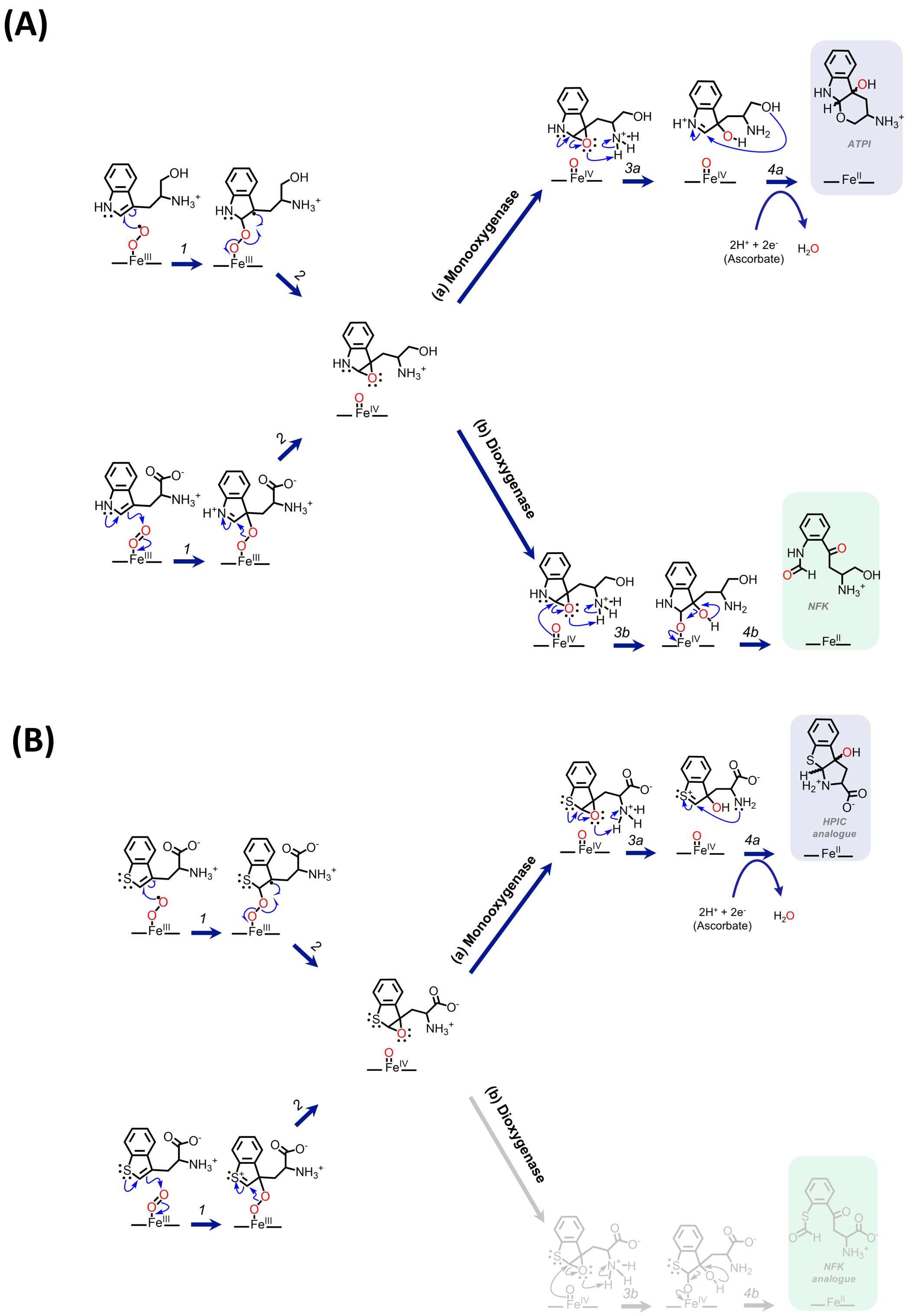

Figure S17 Proposed Mechanism for Oxidation by hIDO. (A) L-Tryptophanol and **(B)** S-L-Trp undergo oxidation by hIDO, showing the possible concurrent monooxygenase **(a)** and dioxygenase **(b)** pathways. The monooxygenase pathway **(a)** leads to the formation of the cyclic products (ATPI and HPIC, respectively, for L-tryptophanol and S-L-Trp), while the dioxygenase pathway **(b)** leads to the NFK analogue. For L-tryptophanol both (a) and (b) pathways are used. For S-L-Trp only the monooxygenase pathway is active, while the dioxygenase pathway (leading to the NFK analogue) does not occur and is therefore shown in grey.

4. TABLES

Table S1. ChemPLP docking scores for *cis*- and *trans*-HPIC bound to IDO. The highest fitness scores for each isomer are shown, indicating a consistent preference for *cis*-HPIC binding.

	**L-Trp**		**S- L-Trp**	
**HPIC**	**Docking Solution**	**Fitness Score**	**Docking Solution**	**Fitness Score**
cis	1	61.02	3	70.50
trans	3	42.22	2	44.15

Table S2. ^{1}H and ^{13}C NMR data for synthetic NFK (600 MHz, D_2O/H_2O).

**Compound Structure**	**Atom**	**Proton Chemical Shift (ppm)**	**Carbon Chemical Shift (ppm)**	**Multiplicity**	**J Coupling (Hz)**	**Integration**
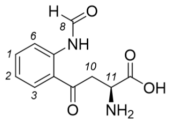	1	7.57	135	t	-	1
	2	7.37	125	t	-	1
	3	7.97(*trans*), 7.91(*cis*)	131(trans), 131(cis)	d, d	7.96, 7.91	0.6, 1
***trans*-NFK**	6	8.17(*cis*), 7.56 (*trans*)	123(*cis*), 118(*trans*)	d, d	8.2, 8.2	1, 0.5
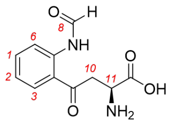	8	8.77(*trans*), 8.34 (*cis*)	165(trans), 163(cis)	s, s	-	0.3, 1
	10	3.6	52	d	3.59	0.5
***cis*-NFK**	11	4.04, 3.66	40, 40	q, m		2.3, 3.5

Table S3. Calculated and experimentally analysed accurate masses of products from hIDO assays with diverse substrates.

**Substrates**	**hIDO activities**	**Molecular formula of products**	**m/z theoretical**	**m/z observed**	**Mass accuracy (ppm)**
L-Trp	Monooxygenase	$C_{11}H_{12}N_2O_3$	221.0921	221.0929	3.6
	Dioxygenase	$C_{11}H_{12}N_2O_4$	237.0870	237.0873	1.3
1-Me-L-Trp	Monooxygenase	$C_{12}H_{14}N_2O_3$	235.1077	-	-
	Dioxygenase	$C_{12}H_{14}N_2O_4$	251.1026	251.1024	-0.8
L-tryptophanol	Monooxygenase	$C_{11}H_{14}N_2O_2$	207.1128	207.1134	3.9
	Dioxygenase	$C_{11}H_{14}N_2O_3$	223.1077	223.1081	1.8
5-OH-Trp	Monooxygenase	$C_{11}H_{12}N_2O_4$	237.0870	-	-
	Dioxygenase	$C_{11}H_{12}N_2O_5$	253.0819	253.0824	2.0
S-L-Trp	Monooxygenase	$C_{11}H_{11}NO_3S$	238.0528	238.0536	3.4
	Dioxygenase	$C_{11}H_{11}NO_4S$	254.0472	-	-

Table S4. ^{1}H and ^{13}C NMR data for synthetic HPIC (600 MHz, D_2O/H_2O).

Compound Structure	Atom	Proton Chemical Shift (ppm)	Carbon Chemical Shift (ppm)	Multiplicity	J Coupling (Hz)	Integration
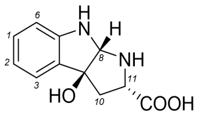 ***trans*-HPIC**	1	7.21	131	m	-	3
	2	6.91	121	dt	-	3
	3	7.24	124	m	-	3
	6	6.75 (*cis*), 6.72(*trans*)	111(*cis*). 111(*trans*)	d, d	6.74, 6.74	2, 1
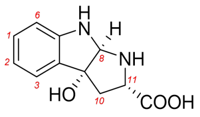 ***cis*-HPIC**	8	5.31(*cis*), 5.22 (*trans*)	84(*cis*), 84(*trans*)	s, s	-	2, 1
	10	2.81, 2.46	41	t, m	-	4, 2
	11	4.24, 3.76	60.5, 60.5	t, t	-	2, 1

5. REFERENCES

(1) Chauhan, N.; Basran, J.; Efimov, I.; Svistunenko, D. A.; Seward, H. E.; Moody, P. C. E.; Raven, E. L. The Role of Serine 167 in Human Indoleamine 2,3-Dioxygenase: A Comparison with Tryptophan 2,3-Dioxygenase. *Biochemistry* **2008**, *47* (16), 4761-4769.

(2) Booth, E. S.; Basran, J.; Lee, M.; Handa, S.; Raven, E. L. Substrate Oxidation by Indoleamine 2,3-Dioxygenase: evidence for a common reaction mechanism. *Journal of Biological Chemistry* **2015**, *290* (52), 30924-30930.

(3) Papadopoulou, N. D.; Mewies, M.; McLean, K. J.; Seward, H. E.; Svistunenko, D. A.; Munro, A. W.; Raven, E. L. Redox and spectroscopic properties of human indoleamine 2,3-dioxygenase and a His303Ala variant: implications for catalysis. *Biochemistry* **2005**, *44* (43), 14318-14328.

(4) Lewis-Ballester, A.; Pham, K. N.; Batabyal, D.; Karkashon, S.; Bonanno, J. B.; Poulos, T. L.; Yeh, S. R. Structural insights into substrate and inhibitor binding sites in human indoleamine 2,3-dioxygenase 1. *Nat Commun* **2017**, *8* (1), 1693.

(5) Waterhouse, A.; Bertoni, M.; Bienert, S.; Studer, G.; Tauriello, G.; Gumienny, R.; Heer, F. T.; de Beer, T. A P.; Rempfer, C.; Bordoli, L.; et al. SWISS-MODEL: homology modelling of protein structures and complexes. *Nucleic Acids Research* **2018**, *46* (W1), W296-W303.

(6) Anandakrishnan, R.; Aguilar, B.; Onufriev, A. V. H++ 3.0: automating pK prediction and the preparation of biomolecular structures for atomistic molecular modeling and simulations. *Nucleic Acids Res* **2012**, *40* (Web Server issue), W537-541.

(7) Tian, C.; Kasavajhala, K.; Belfon, K. A. A.; Raguette, L.; Huang, H.; Migues, A. N.; Bickel, J.; Wang, Y.; Pincay, J.; Wu, Q.; et al. ff19SB: Amino-Acid-Specific Protein Backbone Parameters Trained against Quantum Mechanics Energy Surfaces in Solution. *J Chem Theory Comput* **2020**, *16* (1), 528-552.

(8) Horn, A. H. A consistent force field parameter set for zwitterionic amino acid residues. *J Mol Model* **2014**, *20* (11), 2478.

(9) Wang, J.; Wolf, R. M.; Caldwell, J. W.; Kollman, P. A.; Case, D. A. Development and testing of a general amber force field. *J Comput Chem* **2004**, *25* (9), 1157-1174.

(10) Jakalian, A.; Jack, D. B.; Bayly, C. I. Fast, efficient generation of high-quality atomic charges. AM1-BCC model: II. Parameterization and validation. *J Comput Chem* **2002**, *23* (16), 1623-1641.

(11) Abu Hassan, A.; Hanževački, M.; Pordea, A. Computational investigation of cis-1,4-polyisoprene binding to the latex-clearing protein LcpK30. *PLoS One* **2024**, *19* (5), e0302398.

(12) Li, P.; Merz, K. M., Jr. MCPB.py: A Python Based Metal Center Parameter Builder. *J Chem Inf Model* **2016**, *56* (4), 599-604.

(13) Izadi, S.; Anandakrishnan, R.; Onufriev, A. V. Building Water Models: A Different Approach. *J Phys Chem Lett* **2014**, *5* (21), 3863-3871.

(14) Sengupta, A.; Li, Z.; Song, L. F.; Li, P.; Merz, K. M., Jr. Parameterization of Monovalent Ions for the OPC3, OPC, TIP3P-FB, and TIP4P-FB Water Models. *J Chem Inf Model* **2021**, *61* (2), 869-880.

(15) Åqvist, J.; Wennerström, P.; Nervall, M.; Bjelic, S.; Brandsdal, B. O. Molecular dynamics simulations of water and biomolecules with a Monte Carlo constant pressure algorithm. *Chemical Physics Letters* **2004**, *384* (4), 288-294.

(16) Kräutler, V.; Van Gunsteren, W. F.; Hünenberger, P. H. A fast SHAKE:: Algorithm to solve distance constraint equations for small molecules in molecular dynamics simulations. *Journal of computational chemistry* **2001**, *22* (5), 501-508.
(17) Case, D.; Aktulga, H. M.; Belfon, K.; Ben-Shalom, I.; Brozell, S.; Cerutti, D.; Cheatham, T.; Cisneros, G. A.; Cruzeiro, V.; Darden, T.; et al. *Amber 2022*; 2022.
(18) DeLano, W. L. The PyMol Molecular Graphis System. *DeLano Scientific, San Carlos, CA, USA* **2002**.
(19) Frisch, M. J.; Trucks, G. W.; Schlegel, H. B.; Scuseria, G. E.; Robb, M. A.; Cheeseman, J. R.; Scalmani, G.; Barone, V.; Petersson, G. A.; Nakatsuji, H.; et al. *Gaussian 16. Revision C.01* **2016**, Gaussian, Inc.: Wallingford, CT.
(20) Grimme, S. Exploration of Chemical Compound, Conformer, and Reaction Space with Meta-Dynamics Simulations Based on Tight-Binding Quantum Chemical Calculations. *J Chem Theory Comput* **2019**, *15* (5), 2847-2862.
(21) Pracht, P.; Bohle, F.; Grimme, S. Automated exploration of the low-energy chemical space with fast quantum chemical methods. *Physical Chemistry Chemical Physics* **2020**, *22* (14), 7169-7192, 10.1039/C9CP06869D.
(22) Bannwarth, C.; Caldeweyher, E.; Ehlert, S.; Hansen, A.; Pracht, P.; Seibert, J.; Spicher, S.; Grimme, S. Extended tight-binding quantum chemistry methods. *Wiley Interdisciplinary Reviews: Computational Molecular Science* **2021**, *11* (2), e1493.
(23) Lu, T.; Chen, F. Multiwfn: a multifunctional wavefunction analyzer. *Journal of computational chemistry* **2012**, *33* (5), 580-592.
(24) Humphrey, W.; Dalke, A.; Schulten, K. VMD: visual molecular dynamics. *Journal of molecular graphics* **1996**, *14* (1), 33-38.
(25) Weinhold, F.; Landis, C. R. Natural Bond Orbitals and Extensions of Localized Bonding Concepts. *Chem. Educ. Res. Pract.* **2001**, *2*, 91-104.
(26) Glendening, E. D.; Reed, A. E.; Carpenter, J. E.; Weinhold, F., NBO Version 3.1.
(27) Jones, G.; Willett, P.; Glen, R. C.; Leach, A. R.; Taylor, R. Development and validation of a genetic algorithm for flexible docking. *Journal of molecular biology* **1997**, *267* (3), 727-748.
(28) Korb, O.; Stutzle, T.; Exner, T. E. Empirical scoring functions for advanced protein-ligand docking with PLANTS. *J Chem Inf Model* **2009**, *49* (1), 84-96.
